# On the synchronization techniques of chaotic oscillators and their FPGA-based implementation for secure image transmission

**DOI:** 10.1371/journal.pone.0209618

**Published:** 2019-02-06

**Authors:** Omar Guillén-Fernández, Ashley Meléndez-Cano, Esteban Tlelo-Cuautle, Jose Cruz Núñez-Pérez, Jose de Jesus Rangel-Magdaleno

**Affiliations:** 1 Department of Electronics, INAOE, Tonantzintla, Puebla, Mexico; 2 Instituto Politécnico Nacional, IPN-CITEDI, Av. Instituto Politécnico Nacional No. 1310, Tijuana BC, 22435 Mexico; Shandong University of Science and Technology, CHINA

## Abstract

Synchronizing chaotic oscillators has been a challenge to guarantee successful applications in secure communications. That way, three synchronization techniques are applied herein to twenty two chaotic oscillators, three of them based on piecewise-linear functions and nineteen proposed by Julien C. Sprott. These chaotic oscillators are simulated to generate chaotic time series that are used to evaluate their Lyapunov exponents and Kaplan-Yorke dimension to rank their unpredictability. The oscillators with the high positive Lyapunov exponent are implemented into a field-programmable gate array (FPGA), and afterwards they are synchronized in a master-slave topology applying three techniques: the seminal work introduced by Pecora-Carroll, Hamiltonian forms and observer approach, and open-plus-closed-loop (OPCL). These techniques are compared with respect to their synchronization error and latency that is associated to the FPGA implementation. Finally, the chaotic oscillators providing the high positive Lyapunov exponent are synchronized and applied to a communication system with chaotic masking to perform a secure image transmission. Correlation analysis is performed among the original image, the chaotic channel and the recovered image for the three synchronization schemes. The experimental results show that both Hamiltonian forms and OPCL can recover the original image and its correlation with the chaotic channel is as low as 0.00002, demonstrating the advantage of synchronizing chaotic oscillators with high positive Lyapunov exponent to guarantee high security in data transmission.

## 1 Introduction

Secure communication systems have been developed since the introduction of the first synchronization approach between two chaotic oscillators by Pecora and Carroll [1, 2]. Nowadays, several challenges remain open to accomplish and guarantee privacy and high security of the transmitted information, so that researchers are searching for the best chaotic oscillator and synchronization approaches. For example, the authors in [3] revised 50 chaotic oscillators to demonstrate that their proposed new chaotic one is much better for security applications. However, they did not optimize the positive Lyapunov exponent to rank the unpredictability of the 50 oscillators. Other authors have been introduced novel chaotic secure communication systems [4] highlighting the necessity of a good synchronization approach in order to guarantee a successful transmission of information. In addition, those contributions must accomplish the requirements imposed by the modern cryptographic applications in the industrial internet of things [5] and wireless sensor networks [6], to provide privacy and security.

During the last years, many synchronization techniques for chaotic oscillators have been introduced along with some applications to security, see for example the recent works given in [7–17]. The main objective of synchronizing two chaotic oscillators is oriented to develop secure communication systems to preserve privacy, provide security and be robust to attacks. These issues can be accomplished using chaotic oscillators because they have the property of high sensitivity to the initial conditions, which can be quantified by evaluating and maximizing the positive Lyapunov exponent. The evaluation of the fractal dimension also provides characteristics to rank the randomness and unpredictability of chaotic oscillators. In this manner, this work shows that the master-slave synchronization of two chaotic oscillators having high positive Lyapunov exponents guarantees high security, and if the synchronization error is very low then the original information can be recovered without loss of data. This can be proved through evaluating the correlation among the original data, the chaotic channel that masks the information being transmitted with chaos, and the recovered data. This is not a trivial task because if one does not choose a good numerical method with a good step size, then the simulation may induce artificial chaos suppression or can engender the appearance of spurious solutions. This is shown in [18], where the authors compare one-step methods suitable for field-programmable gate array (FPGA) implementation versus a method based on trigonometric polynomials, concluding that the last one is ad hoc to solve dynamical systems with oscillatory characteristics. In this work we choose the correct step size of the numerical method by evaluating the equilibrium points and then the eigenvalues of 22 chaotic oscillators, three of them based on piecewise-linear functions (PWL) and nineteen proposed by Julien C. Sprott [19–21]. The positive Lyapunov exponent is evaluated from chaotic time series and then the synchronization is performed between two chaotic oscillators in a master-slave topology.

The article is organized as follows: Section 2 shows the mathematical models and simulation of three chaotic oscillators based on PWL functions and nineteen proposed by Sprott [19]. The equilibrium points and the eigenvalues of the 22 chaotic oscillators are computed as well as the positive Lyapunov exponent of each chaotic time serie, and their respective Kaplan-York dimension. Section 3 details three synchronization techniques, namely: the seminal work of Pecora-Carroll [1], the seminal work on Hamiltonian forms and observer approach [22], and the open-plus-closed-loop (OPCL) technique [23]. These techniques are compared according to their synchronization error. Section 4 shows experimental results of the chaotic attractors implemented in an FPGA, the synchronization results between two chaotic oscillators in a master-slave topology, and the transmission of an image through chaotic masking using the FPGA-based implementation of a secure communication system. Finally, the conclusions are summarized in Sect. 5.

## 2 Chaotic oscillators

Among all kinds of chaotic oscillators, we choose three mathematical models based on PWL functions [24–27], and nineteen proposed by Julien Sprott [20].

**Fig 1:**
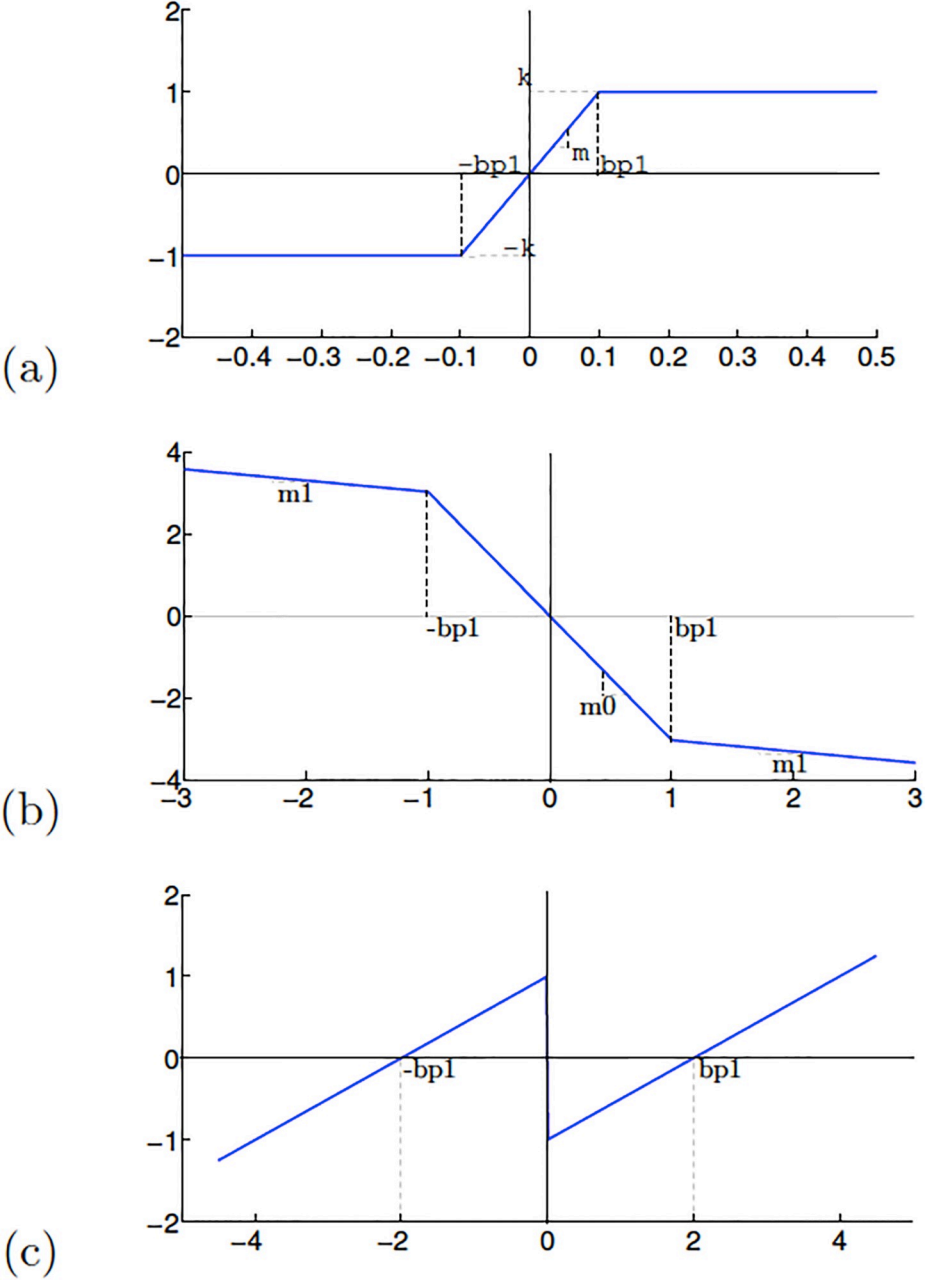
PWL function in a chaotic oscillator based on: (a) SNLF series, (b) negatives slopes, and (c) sawtooth function.

Lets us consider the chaotic oscillator based on saturated nonlinear function (SNLF) series that is modeled by (1). It consists of three state variables *x*_1_, *x*_2_, *x*_3_, four coefficients *a*, *b*, *c*, *d*_1_ and the SNLF *f*(*x*_1_) that can be approached by the PWL function described by (2) and shown in Fig 1(a) to generate 2-scrolls. In (2), *bp*1 denotes the breakpoints, and *f*(*x*_1_) can be increased to generate multi-scroll attractors, as detailed in [26].

$$\begin{array}{c} \begin{array}{cc} & \dot{x_{1}}=x_{2}\\ & \dot{x_{2}}=x_{3}\\ & \dot{x_{3}}=-ax_{1}-bx_{2}-cx_{3}+d_{1}f\left(x_{1}\right) \end{array} \end{array}$$ (1)

$$\begin{array}{c} f\left(x_{1}\right)=\{\begin{array}{cc} -k, & \text{si}\;x_{1}<-bp1\\ mx_{1}, & \text{si}\;-bp1\leq x_{1}\leq bp1\\ k, & \text{si}\;x_{1}>bp1 \end{array} \end{array}$$ (2)

Eq (3) shows another chaotic oscillator having three coefficients (*α*, *β*, *γ*) and a PWL function *f*(*x*_1_). It can be described by (4) and sketched in Fig 1(b), where one can see two negative slopes *m*_0_ and *m*_1_ to generate 2-scrolls. The slopes increase as well as the break points to generate multi-scroll chaotic attractors.

$$\begin{array}{c} \begin{array}{cc} & \dot{x_{1}}=\alpha \left(x_{2}-x_{1}-f\left(x_{1}\right)\right)\\ & \dot{x_{2}}=\gamma \left(x_{1}-x_{2}+x_{3}\right)\\ & \dot{x_{3}}=-\beta x_{2} \end{array} \end{array}$$ (3)

$$\begin{array}{c} f\left(x_{1}\right)=m_{2n-1}x_{1}+\frac{1}{2}\sum_{i-q}^{2n+1}\left(m_{i-1}-m_{i}\right)\left(\left|x_{1}+bp_{i}\right|-\left|x_{1}-bp_{i}\right|\right) \end{array}$$ (4)

The chaotic oscillator described by (5) consists of three coefficients (*α*, *β*, *γ*) and *f*(*x*_1_), which can be approached by the PWL function given in (6) and sketched by Fig 1(c). Also, increasing the slopes as well as the break points one can generate multi-scroll chaotic attractors.

$$\begin{array}{c} \begin{array}{cc} & \dot{x_{1}}=\alpha x_{2}+f\left(x_{1}\right)\\ & \dot{x_{2}}=\alpha x_{1}-\gamma x_{2}-\alpha x_{3}\\ & \dot{x_{3}}=\beta x_{2} \end{array} \end{array}$$ (5)

$$\begin{array}{c} \begin{array}{cc} & f\left(x_{1}\right)=\xi \left(Bp\cdot sgn\left(x_{1}\right)\cdot \left(2k-1\right)-x_{1}\right)\\ & \;\text{si}\;2kBp\geq \left|x_{1}\right|>2\left(k-1\right)Bp \end{array} \end{array}$$ (6)

Another kind of chaotic oscillators are the ones proposed by Sprott [20], which are based in the multiplication of state variables. Sprott took as reference the Poincare-Bendixson theorem [28], and then from (7) he discovered and selected the nineteen listed in Table 1.

$$\begin{array}{c} \begin{array}{cc} & \dot{x}=a+\sum_{i=1}^{3}b_{i}x_{i}+\sum_{i=1}^{3}\sum_{j=1}^{3}c_{i,j}x_{i}x_{j} \end{array} \end{array}$$ (7)

**Table 1:** 19 chaotic oscillators proposed by J. C. Sprott [20].

Case	Equations		
*A*	$\dot{x_{1}}=x_{2}$	$\dot{x_{2}}=-x_{1}+x_{2}x_{3}$	$\dot{x_{3}}=1-x_{2}^{2}$
*B*	$\dot{x_{1}}=x_{2}x_{3}$	$\dot{x_{2}}=x_{1}-x_{2}$	$\dot{x_{3}}=1-x_{1}x_{2}$
*C*	$\dot{x_{1}}=x_{2}x_{3}$	$\dot{x_{2}}=x_{1}-x_{2}$	$\dot{x_{3}}=1-x_{1}^{2}$
*D*	$\dot{x_{1}}=-x_{2}$	$\dot{x_{2}}=x_{1}+x_{3}$	$\dot{x_{3}}=x_{1}x_{3}+3x_{2}^{2}$
*E*	$\dot{x_{1}}=x_{2}x_{3}$	$\dot{x_{2}}=x_{1}^{2}-x_{2}$	$\dot{x_{3}}=1-4x_{1}$
*F*	$\dot{x_{1}}=x_{2}+x_{3}$	$\dot{x_{2}}=-x_{1}+\frac{1}{2}x_{2}$	$\dot{x_{3}}=x_{1}^{2}-x_{3}$
*G*	$\dot{x_{1}}=0.4x_{1}+x_{3}$	$\dot{x_{2}}=x_{1}x_{3}-x_{2}$	$\dot{x_{3}}=-x_{1}+x_{2}$
*H*	$\dot{x_{1}}=-x_{2}+x_{3}^{2}$	$\dot{x_{2}}=x_{1}+\frac{1}{2}x_{2}$	$\dot{x_{3}}=x_{1}-x_{3}$
*I*	$\dot{x_{1}}=-0.2x_{2}$	$\dot{x_{2}}=x_{1}+x_{3}$	$\dot{x_{3}}=x_{1}+x_{2}^{2}-x_{3}$
*J*	$\dot{x_{1}}=2x_{3}$	$\dot{x_{2}}=-2x_{2}+x_{3}$	$\dot{x_{3}}=-x_{1}+x_{2}+x_{2}^{2}$
*K*	$\dot{x_{1}}=x_{1}x_{2}-x_{3}$	$\dot{x_{2}}=x_{1}-x_{2}$	$\dot{x_{3}}=x_{1}+0.3x_{3}$
*L*	$\dot{x_{1}}=x_{2}+3.9x_{3}$	$\dot{x_{2}}=0.9x_{1}^{2}-x_{2}$	$\dot{x_{3}}=1-x_{1}$
*M*	$\dot{x_{1}}=-x_{3}$	$\dot{x_{2}}=x_{1}^{2}-x_{2}$	$\dot{x_{3}}=1.7+1.7x_{1}+x_{2}$
*N*	$\dot{x_{1}}=-2x_{2}$	$\dot{x_{2}}=x_{1}+x_{3}^{2}$	$\dot{x_{3}}=1+x_{2}-2x_{3}$
*O*	$\dot{x_{1}}=x_{2}$	$\dot{x_{2}}=x_{1}-x_{3}$	$\dot{x_{3}}=x_{1}+x_{1}x_{3}+2.7x_{2}$
*P*	$\dot{x_{1}}=2.7x_{2}+x_{3}$	$\dot{x_{2}}=-x_{1}+x_{2}^{2}$	$\dot{x_{3}}=x_{1}+x_{2}$
*Q*	$\dot{x_{1}}=-x_{3}$	$\dot{x_{2}}=x_{1}-x_{2}$	$\dot{x_{3}}=3.1x_{1}+x_{2}^{2}+0.5x_{3}$
*R*	$\dot{x_{1}}=0.9-x_{2}$	$\dot{x_{2}}=0.4+x_{3}$	$\dot{x_{3}}=x_{1}x_{2}-x_{3}$
*S*	$\dot{x_{1}}=-x_{1}-4x_{2}$	$\dot{x_{2}}=x_{1}+x_{3}^{2}$	$\dot{x_{3}}=1+x_{1}$

From the mathematical models of the 22 chaotic oscillators described above, one must analyze their static and dynamical properties. That way, as detailed by Sprott [20], Tables 2 and 3 list the equilibrium points and eigenvalues of the chaotic oscillators based on the three PWL functions and the Sprott collection.

**Table 2:** Equilibrium points and eigenvalues of the three chaotic oscillators based on PWL functions.

*Oscillator type*	*Function*	*Equilibrium Points*	*Eigenvalues*		
			λ_1_	λ_2_	λ_3_
***PWL Function***	***SNLF Series***	EP1(-1,0,0)	-0.84	0.074 − 0.90i	0.074 + 0.90i
		EP2(0,0,0)	3.18	-1.943 + 3.05i	-1.943 − 3.05i
		EP3(0,0,0)	-0.84	0.074 − 0.90i	0.074 + 0.90i
	***Negative Slopes***	EP1(3,0,3)	2.37	-2.403 − 1.67i	-2.403 + 1.67i
		EP2(0,0,0)	-3.50	2.680 − 2.11i	2.680 + 2.11i
		EP3(3,0,3)	2.37	-2.403 − 1.67i	-2.403 + 1.67i
	***Sawtooth Function***	EP1(-1,0,-1)	1.73	-0.965 − 2.14i	-0.965 + 2.14i
		EP2(-1,0,-1)	0	-0.5 − 1.65i	-0.5 + 1.65i
		EP3(1,0,1)	1.73	-0.965 + 2.14i	-0.965 − 2.14i

**Table 3:** Equilibrium points and eigenvalues of Sprott’s collection.

*Oscillator Type*	*Case*	*Equilibrium Points*	*Eigenvalues*		
			λ_1_	λ_2_	λ_3_
***Sprott’s Collection***	A	Undefined	Undefined	Undefined	Undefined
	B	EP1(-1,-1,0) EP2(1,1,0)	-1.353	0.176 + 1.202i	0.176 − 1.202i
		EP2(1,1,0)			
	C	EP1(-1,-1,0)	-1	0 + 1.414i	0 − 1.414i
		EP2(1,1,0)			
	D	EP1(0,0,0)	0	i	- i
	E	EP1(0.25,0.062,0)	-1	0.5i	- 0.5i
	F	EP1(-2,-4,4)	0.214	- 0.357 + 2.12i	- 0.357 − 2.12i
		EP2(0,0,0)	-1	0.25 + 0.96i	0.25 − 0.96i
	G	EP1(-2.5,-2.5,1)	- 1	- 0.2 + 0.979i	- 0.2 − 0.979i
		EP2(0,0,0)	- 0.297	-0.448+1.779i	-0.448−1.779i
	H	EP1(-2,4,-2)	0.214	- 0.357 + 2.12i	- 0.357 − 2.12i
		EP2(0,0,0)	-1	0.25 + 0.96i	0.25 − 0.96i
	I	EP1(0,0,0)	-1.134	0.067 + 0.59i	0.067 − 0.59i
	J	EP1(0,0,0)	- 2.314	0.157 + 1.305i	0.157−1.305i
	K	EP1(0,0,0)	-1	0.15 + 0.98i	0.15 − 0.98i
		EP2(-3.3,-3.3,11.1)	0.143	- 2.088 + 1.61i	- 2.088 − 1.61i
	L	EP1(1,0.9,-0.23)	- 1.433	0.216 + 1.635i	0.216 − 1.635i
	M	EP1(2.4,-5.79,0)	0.907	- 0.953 + 1.58i	- 0.953 − 1.58i
		EP2(-0.7,-0.49,0)	- 1.389	0.194 + 1.48i	0.194 − 1.48i
	N	EP1(-0.25,0,0.5)	- 2.314	0.157 + 1.305i	0.157 − 1.305i
	O	EP1(0,0,0)	-0.51	0.255+1.37i	0.255−1.37i
		EP2(-1,0,-1)	0.431	-0.715 + 1.34i	-0.715−1.34i
	P	EP1(0,0,0)	-0.510	0.255 + 1.37i	0.255 − 1.37i
		EP2(1,-1,2.7)	0.382	-1.191 + 1.09i	- 1.191 − 1.09i
	Q	EP1(0,0,0)	-1	0.25 + 1.742i	0.25 − 1.742i
		EP2(-3.1,-3.1,0)	0.834	-0.667 + 1.80i	-0.667 − 1.80i
	R	EP1(-0.44,0.9,-0.4)	-1.232	0.116 + 0.84i	0.116 − 0.84i
	S	EP1(-1,0.25,-1)	1.202	-1.101 + 2.33i	-1.101 − 2.33i
		EP2(-1,0.25,1)	-1.607	0.303 + 2.210i	0.303 − 2.210i

The eigenvalues help us to estimate the step size of the numerical method [18], in order to simulate and generate chaotic time series for each state variable of the 22 chaotic oscillators. Therefore, the 66 time series are analyzed to evaluate their associated Lyapunov exponent, which determines the system unpredictability. This task is performed using the free-software “TISEAN 3.0.1”, which can be used within MATLAB. The results are listed in Table 4, where it can be seen the associated Lyapunov exponent and Kaplan-Yorke dimension. Analyzing those results, it can be observed that the chaotic oscillator based on SNLF series, and Sprott’s cases G and L provide the high positive Lyapunov exponent values.

**Table 4:** Lyapunov exponent and fractal dimension of each state variable *x*_1_, *x*_2_, *x*_3_ of the twenty two chaotic oscillators computed by “TISEAN 3.0.1”.

*Oscillator Type*	*Function*	*Lyapunov Exponent*			*Kaplan-Yorke Dimension*		
		*x_1_*	*x_2_*	*x_3_*	*x_1_*	*x_2_*	*x_3_*
***Based on PWL function***	SNLF	9.13e-2	4.22e-2	4.53e-2	2.84	2.78	2.71
	Neg. Slopes	1.44e-2	2.80e-2	1.41e-2	2.28	2.63	2.33
	Sawtooth	2.32e-2	1.42e-2	1.53e-2	2.59	2.31	2.54
***Sprott’s Collection***	A	1.23e-2	1.14e-2	3.02e-2	2.91	2.96	2.74
	B	1.46e-2	2.74e-2	5.21e-2	2.39	2.74	2.66
	C	1.54e-2	3.90e-2	8.21e-2	2.66	2.72	2.83
	D	2.05e-2	2.69e-2	1.35e-2	2.96	2.93	2.93
	E	1.21e-1	1.71e-2	1.02e-1	2.73	2.92	2.90
	F	1.16e-2	7.94e-3	1.89e-2	2.67	2.71	3.00
	G	1.84e-1	1.67e-1	2.33e-1	2.84	2.88	2.88
	H	2.02e-2	1.09e-2	1.37e-2	2.76	2.58	2.72
	I	1.28e-1	1.52e-1	5.44e-2	3.00	2.91	2.92
	J	4.40e-2	6.01e-2	6.64e-2	3.00	2.72	2.92
	K	1.27e-1	1.17e-1	5.85e-2	2.86	2.72	2.93
	L	1.95e-1	1.76e-1	1.87e-1	2.86	2.85	3.00
	M	4.95e-2	2.36e-2	3.39e-2	2.79	2.78	2.84
	N	5.72e-2	6.69e-2	6.06e-2	3.00	2.86	2.72
	O	9.77e-3	2.27e-2	2.29e-2	2.78	2.86	2.85
	P	1.70e-2	1.41e-2	1.14e-2	2.75	2.67	2.71
	Q	1.88e-2	2.58e-3	1.96e-2	2.69	2.33	2.78
	R	1.92e-2	2.01e-1	2.00e-1	2.84	2.93	2.89
	S	1.60e-2	1.72e-2	5.63e-3	2.60	2.52	2.37

The Lyapunov exponents are also evaluated herein by applying “Wolf’s algorithm” [29], which is available in: https://la.mathworks.com/matlabcentral/fileexchange/4628-calculation-lyapunov-exponents-for-ode. In this case, the mathematical model of each chaotic oscillator is used to evaluate the three Lyapunov exponents, the negative, the zero, and the positive one. The results are listed in Table 5 along their corresponding Kaplan-Yorke dimension. As one can see, the chaotic oscillator based on Negative Slopes, and Sprott’s cases B and S provide the high positive Lyapunov exponent values.

**Table 5:** Lyapunov exponents and fractal dimension of the twenty two chaotic oscillators avaluated by “Wolf’s algorithm”.

*Oscillator Type*	*Function*	*Lyapunov Exponents*			*Kaplan-Yorke Dimension*
		λ_+_	λ_0_	λ_−_	
***Based on PWL function***	SNLF	0.099	-0.002	-0.796	2.122
	Neg. Slopes	0.353	0.000	-3.209	2.103
	Sawtooth	0.140	0.108	-1.991	2.125
***Sprott’s Collection***	A	0.001	0.000	-0.001	3.000
	B	0.209	0.000	-1.208	2.172
	C	0.159	0.000	-1.158	2.136
	D	0.103	0.001	-1.314	2.079
	E	0.081	0.000	-1.082	2.075
	F	0.120	0.000	-0.621	2.195
	G	0.036	0.000	-0.637	2.058
	H	0.114	0.000	-0.615	2.187
	I	0.013	0.000	-1.012	2.012
	J	0.073	0.001	-2.074	2.036
	K	0.039	0.000	-0.890	2.043
	L	0.064	0.000	-1.065	2.061
	M	0.043	-0.001	-1.042	2.040
	N	0.077	0.001	-2.078	2.037
	O	0.051	0.000	-0.321	2.161
	P	0.095	0.000	-0.488	2.195
	Q	0.102	0.000	-0.602	2.170
	R	0.060	0.000	-1.061	2.057
	S	0.187	0.000	-1.187	2.157

## 3 Synchronization of two chaotic oscillators in a “master-slave” topology

The synchronization of two chaotic oscillators in a master-slave topology occurs when the trajectories of both state variables meet in the same time with a minimum synchronization error, so that they adjust their behavior temporarily. Among the currently available synchronization techniques for chaotic oscillators, we apply three, namely: the seminal work of Pecora-Carroll, Hamiltonian forms and observer approach, and OPCL.

### 3.1 Pecora-Carroll synchronization technique

**Fig 2:**
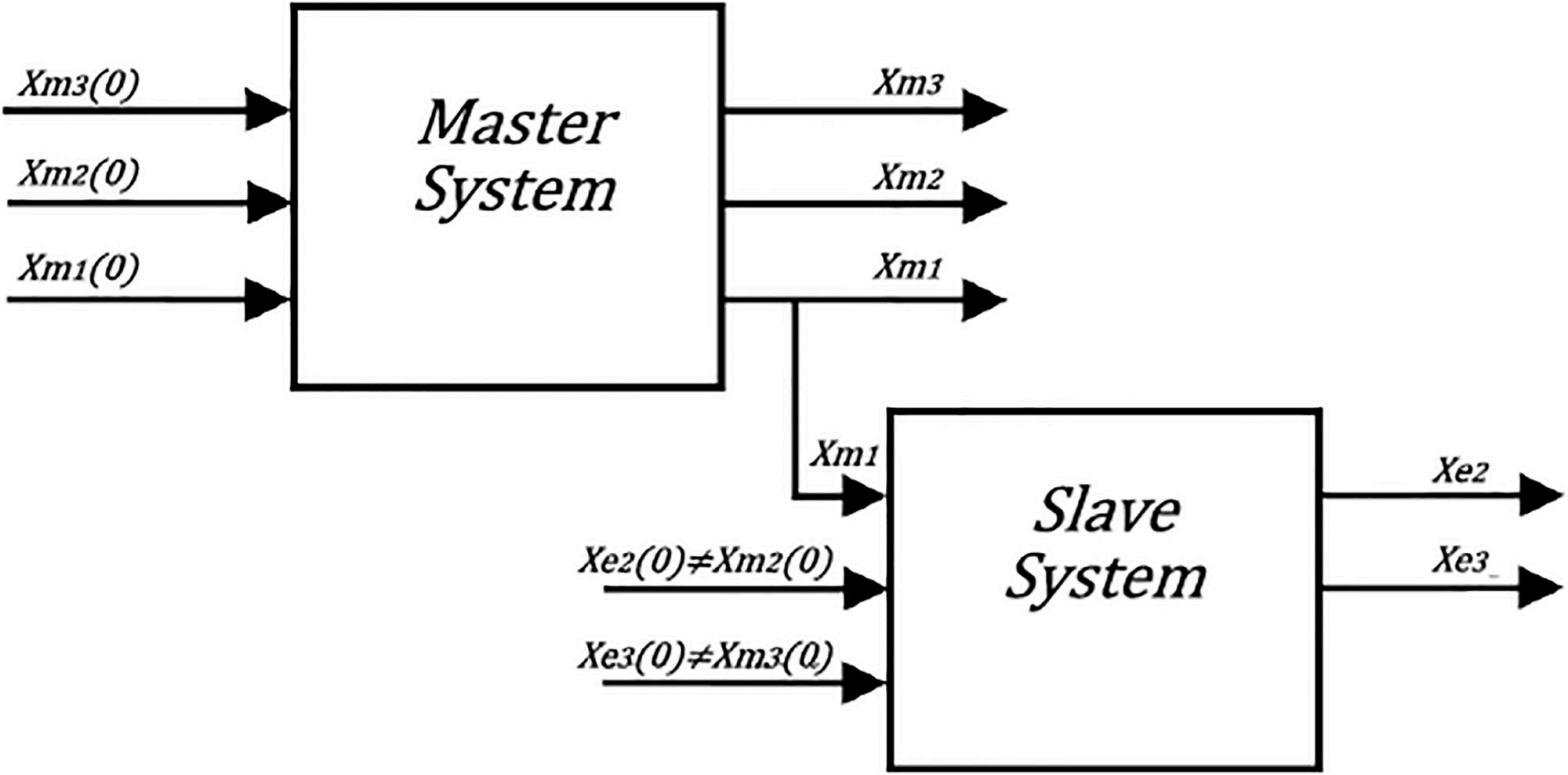
Block diagram of the master-slave chaotic synchronization applying Pecora-Carroll technique by using the state variable *x*_1_ as the driving signal.

This synchronization technique consists of two identical chaotic oscillators [30], with the same parameter values, but evolving in time from different initial conditions. In order to synchronize two chaotic oscillators, the output from, at least, one of the coupled differential equations of the master chaotic oscillator must be made available to the slave chaotic oscillator, as sketched in Fig 2 [31]. To achieve the synchronization one can take any of the three state variables *x*_1_, *x*_2_, *x*_3_ as driving, from the master system. The recommendation to the correct selection of the driver variable is the observation and determination of the influence that it has over the differential equations.

With the topology defined for the synchronization as the master-slave system, the pattern that indicates that the system will be synchronized are the eigenvalues of the Jacobian of the slave system. If the real parts of eigenvalues are negative, the master-slave synchronization will be successful. This is a necessary condition but not enough since there may be a system with eigenvalues equal to zero and the synchronization can occur.

Lets us consider the chaotic oscillator from case L of Sprott’s collection [20], given again in (8), where *m* denotes master. If one considers *x*_1_ as driving because it is the state variable that is present throughout the system and has an influence on the state variables *x*_2_ and *x*_3_, then the slave system can be described by (9).

$$\begin{array}{c} \begin{array}{ccc} \dot{x_{1m}} & = & x_{2m}+3.9x_{3m}\\ \dot{x_{2m}} & = & 0.9x_{1m}^{2}-x_{2m}\\ \dot{x_{3m}} & = & 1-x_{1m} \end{array} \end{array}$$ (8)

$$\begin{array}{c} \begin{array}{ccc} \dot{x_{2s}} & = & 0.9x_{1m}^{2}-x_{2s}\\ \dot{x_{3s}} & = & 1-x_{1m} \end{array} \end{array}$$ (9)

**Fig 3:**
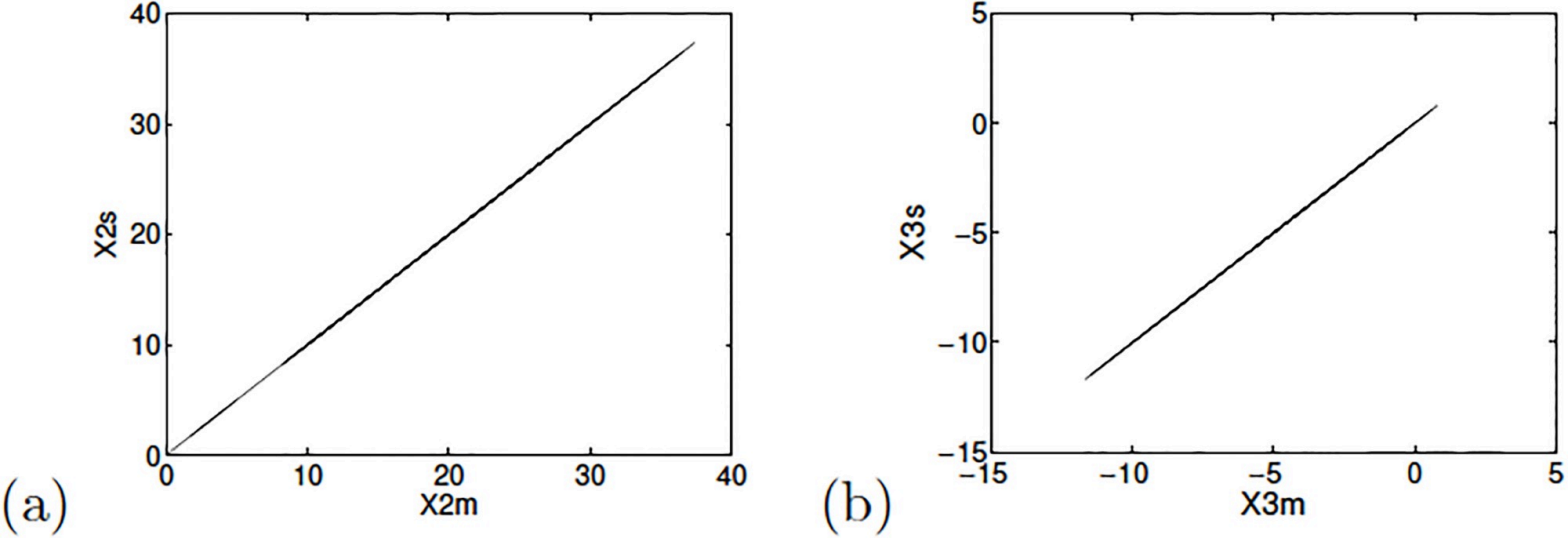
Phase diagrams for the master and slave state variables: (a) *x*_2_ and (b) *x*_3_ for Sprott’s case L.

**Fig 4:**
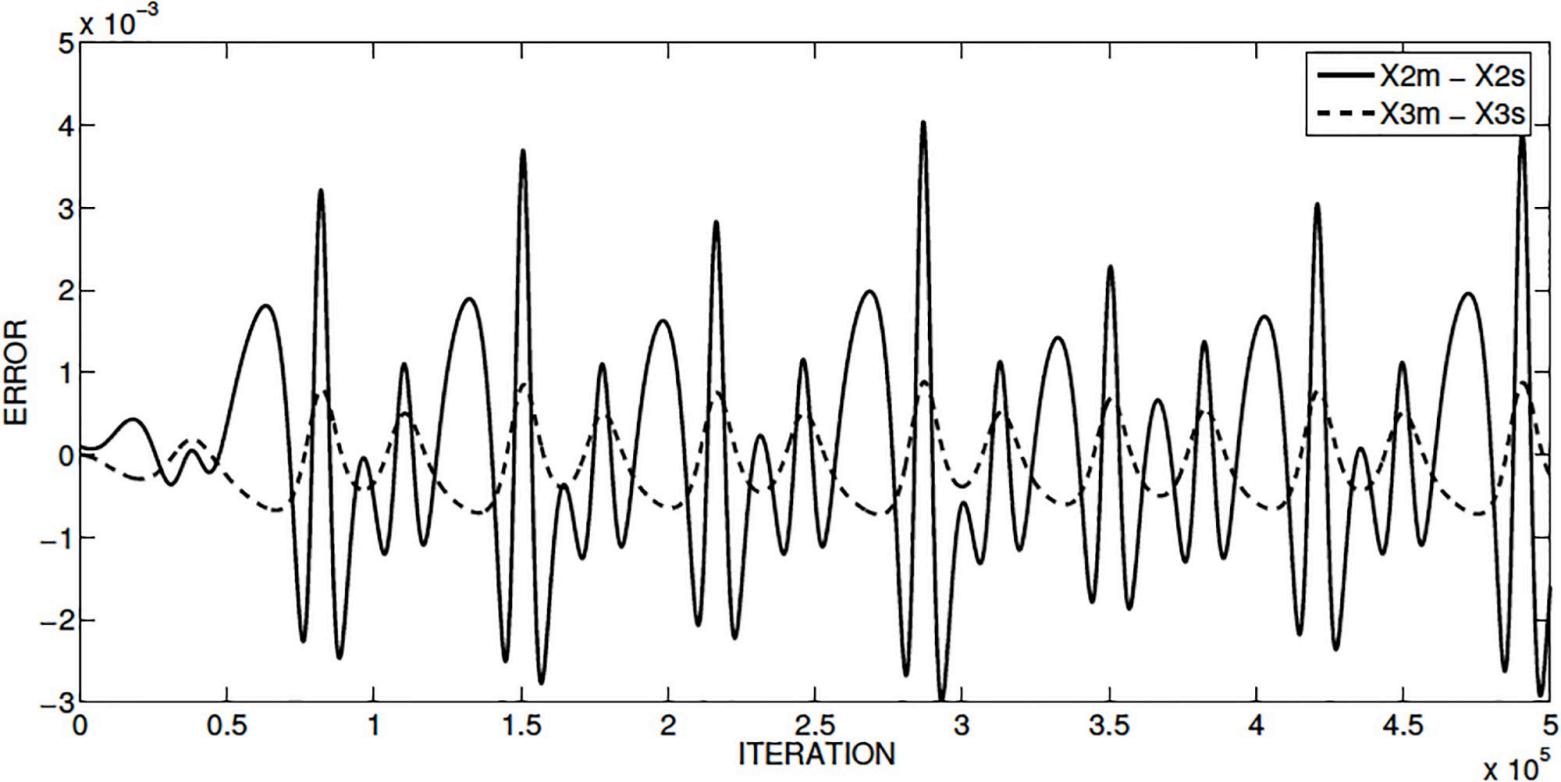
Synchronization error using *x*_1_ as drive for Sprott’s case L, for the master and slave state variables *x*_2_ and *x*_3_.

The evaluation of the Jacobian slave matrix is zero, so that the eigenvalues are: λ_1,2_ = 0. Realizing the simulation of the synchronization using MATLAB, Fig 3 shows the phase synchronization diagrams between the state variables *x*_2_ and *x*_3_. Fig 4 shows the synchronization error for the two state variables between the master and slave oscillators.

### 3.2 Hamiltonian forms and observer approach technique

Every chaotic oscillator can be described by $\dot{x}=f\left(x\right)$, which according to the seminal work given in [22]; the Hamiltonian approach can be described by (10), where ∂*H* is the gradient vector of the energy function *H*, positive definite in *R*^*n*^. *H* is a quadratic function defined by $H\left(x\right)=\frac{1}{2}X^{T}Mx$, with *M* as a symmetrical matrix and positive definite. *J*(*x*) and *S*(*x*) are matrices representing the conservative and non-conservative parts of the system, respectively, and must satisfy: *J*(*x*)+*J*^*T*^(*x*) = 0 and *S*(*x*) = *S*^*T*^(*x*). There exists the possibility to add a destabilizing vector as *F*(*x*), to get the form of a Hamiltonian system, as shown in (11). This can consider suppositions to get the form given in (10), without *F*(*x*).

$$\begin{array}{c} \dot{x}=J\left(x\right)\frac{\partial H}{\partial x}+S\left(x\right)\frac{\partial H}{\partial x},\;x\;\epsilon \;R^{n} \end{array}$$ (10)

$$\begin{array}{c} \dot{x}=J\left(x\right)\frac{\partial H}{\partial x}+S\left(x\right)\frac{\partial H}{\partial x}+F\left(x\right),\;x\;\epsilon \;R^{n} \end{array}$$ (11)

If one considers the system with destabilizing vector and one linear output, one gets (12), where *y* is a vector denoting the output of the system. In addition, if *ξ* is the estimated state vector of *x* and *η* the estimated output in terms of *ξ*, then an observer to (11) can be given by (13), where K is a vector of constant gains.

$$\begin{array}{c} \begin{array}{cc} & \dot{x}=J\left(y\right)\frac{\partial H}{\partial x}+S\left(y\right)\frac{\partial H}{\partial x}+F\left(y\right),\;x\;\epsilon \;R^{n}\\ & y=C\frac{\partial H}{\partial x},\;y\;\epsilon \;R^{m} \end{array} \end{array}$$ (12)

$$\begin{array}{c} \begin{array}{cc} & \dot{\xi }=J\left(y\right)\frac{\partial H}{\partial \xi }+S\left(y\right)\frac{\partial H}{\partial \xi }+F\left(y\right)+K\left(y-\eta \right)\\ & \eta =C\frac{\partial H}{\partial \xi } \end{array} \end{array}$$ (13)

The synchronization by Hamiltonian forms is achieved after accomplishing 2 Theorems:

*Theorem 1: The state x of the nonlinear system* (12) *can be global, exponential and asymptotically estimated by the state of an observer of the form* (13), *if the pair of matrices (C,S) are observables*.

*Theorem 2: The state x of the nonlinear system* (12) *can be global, exponential and asymptotically estimated by the state of an observer of the form* (13), *if and only if there exists a constant matrix K such that the symmetric matrix in* (14) *be negative definite* [22].

$$\begin{array}{cc} \left[W-KC\right]+{\left[W-KC\right]}^{T} & =\left[S-KC\right]+{\left[S-KC\right]}^{T}\\ & =2[S-\frac{1}{2}\left(KC+C^{T}K^{T}\right)] \end{array}$$ (14)

Lets us consider again case L of Sprott’s collection, proposing the master system similar to the original one given in (8) and the energy function as in (15), then the Hamiltonian system given in (16) arises. It becomes the master and the slave system is proposed by adding the gain vector multiplied by the error. The gain vector is obtained verifying that it contains the pair of matrices (*C*, *S*). In this manner, the gain vector *K* can be obtained by applying the Sylvester criterion for negative definite matrices. Herein the gains are equal to *k*_1_ = 1, *k*_2_ = 3, *k*_3_ = 4 and the observer system is described by (17). Finally, the slave system is given in (18),

$$\begin{array}{c} H\left(x\right)=\frac{1}{2}\left[x_{1}^{2}+x_{2}^{2}+x_{3}^{2}\right] \end{array}$$ (15)

$$\begin{array}{c} \begin{array}{cc} & [\begin{array}{c} \dot{x_{1}}\\ \dot{x_{2}}\\ \dot{x_{3}} \end{array}]=[\begin{array}{ccc} 0 & 0.5 & 2.45\\ -0.5 & 0 & 0\\ -2.45 & 20 & 0 \end{array}]\frac{\partial H}{\partial x}+[\begin{array}{ccc} 0 & 0.5 & 1.45\\ 0.5 & -1 & 0\\ 1.45 & 0 & 0 \end{array}]\frac{\partial H}{\partial x}+\\ & \;+[\begin{array}{c} 0\\ 0.9x_{1}^{2}\\ 1 \end{array}] \end{array} \end{array}$$ (16)

$$\begin{array}{c} \begin{array}{cc} & [\begin{array}{c} \dot{x_{1}}\\ \dot{x_{2}}\\ \dot{x_{3}} \end{array}]=[\begin{array}{ccc} 0 & 0.5 & 2.45\\ -0.5 & 0 & 0\\ -2.45 & 20 & 0 \end{array}]\frac{\partial H}{\partial x}+[\begin{array}{ccc} 0 & 0.5 & 1.45\\ 0.5 & -1 & 0\\ 1.45 & 0 & 0 \end{array}]\frac{\partial H}{\partial x}+\\ & \;+[\begin{array}{c} 0\\ 0.9x_{1}^{2}\\ 1 \end{array}]+[\begin{array}{c} 1\\ 3\\ 4 \end{array}]\left(y-\eta \right) \end{array} \end{array}$$ (17)

$$\begin{array}{c} \begin{array}{cc} & \dot{x_{e1}}=x_{e2}+3.9x_{e_{3}}+\left(x_{m1}-x_{e1}\right)\\ & \dot{x_{e2}}=0.9x_{e1}^{2}-x_{e2}+3\left(x_{m2}-x_{e2}\right)\\ & \dot{x_{e3}}=1-x_{e1}+4\left(x_{m3}-x_{e3}\right) \end{array} \end{array}$$ (18)

**Fig 5:**
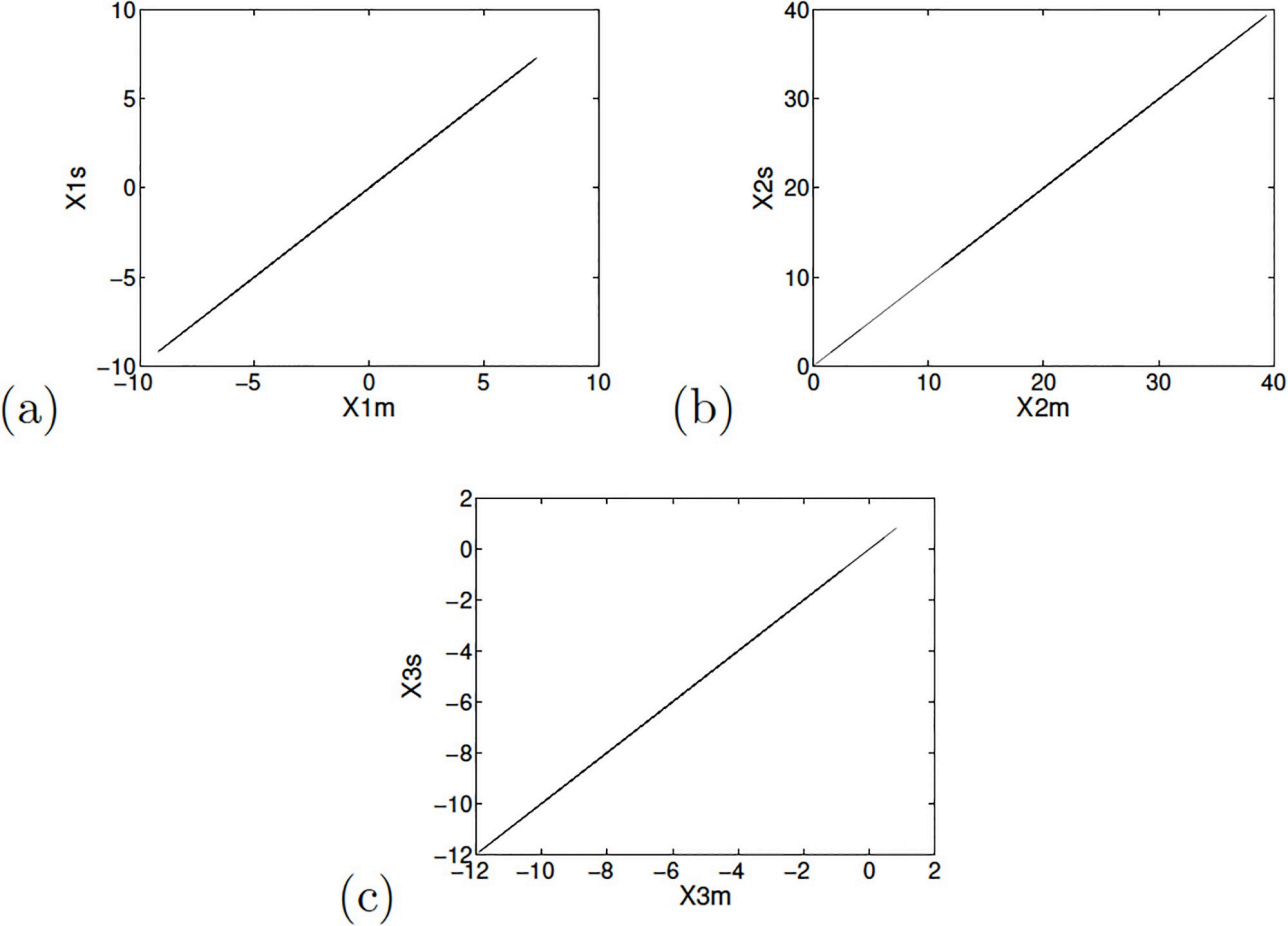
Phase diagrams for the master and slave state variables: (a) *x*_1_, (b) *x*_2_ and (c) *x*_3_ for Sprott’s case L applying Hamiltonian forms and observer approach.

**Fig 6:**
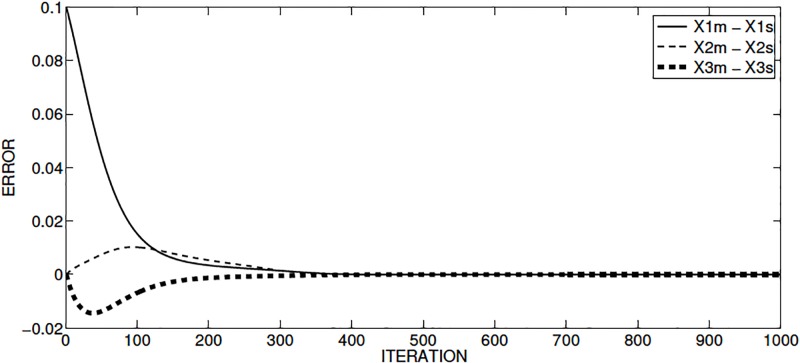
Synchronization error of Sprott’s case L, for the master and slave state variables applying Hamiltonian forms.

The synchronization among the state variables of the master and slave systems is shown in Fig 5. The synchronization error between the master and the slave systems is shown in Fig 6, where it can be seen that the synchronization is accomplished around iteration 800.

### 3.3 OPCL synchronization technique

Open-Plus-Closed-Loop (OPCL) technique is based on the control systems combination. It is a heterogeneous synchronization because allows to obtain the master and slave parameters. From a dynamical system described by $\dot{x}=f\left(x\right)$, the master chaotic oscillator is given by (19), where *x*_*m*1_(*t*), *x*_*m*2_(*t*) and *x*_*m*3_(*t*) denote the state variables, and then *x*_*s*1_(*t*), *x*_*s*2_(*t*) and *x*_*s*3_(*t*) denote the slave chaotic oscillator in (20). *D*(*v*(*t*), *u*(*t*)) is given in (21), with *D*_1_ and *D*_2_ as open loop and closed loop parts, respectively, and given by (22) and (23).

$$\begin{array}{c} \frac{d}{dt}u\left(t\right)=F\left(u\left(t\right)\right)=F\left(x_{m1}\left(t\right),x_{m2}\left(t\right),x_{m3}\left(t\right)\right);\;u\in R^{3} \end{array}$$ (19)

$$\begin{array}{c} \frac{d}{dt}v\left(t\right)=F\left(v\left(t\right)\right)+D\left(v\left(t\right),u\left(t\right)\right);\;v\in R^{3} \end{array}$$ (20)

$$\begin{array}{c} D\left(v\left(t\right),u\left(t\right)\right)=D_{1}\left(u\left(t\right)\right)+D_{2}\left(v\left(t\right),u\left(t\right)\right); \end{array}$$ (21)

$$\begin{array}{c} D_{1}\left(u\left(t\right)\right)=\frac{du(t)}{dt}-F\left(u\left(t\right)\right); \end{array}$$ (22)

$$\begin{array}{c} D_{2}\left(v\left(t\right),u\left(t\right)\right)=(H-\frac{\delta }{\delta t}F\left(u\left(t\right)\right))e\left(t\right) \end{array}$$ (23)

*H* is an arbitrary constant Hurwitz matrix, so that the simplicity of the slave system depends on how this matrix is chosen. Besides, *e*(*t*) = *v*(*t*) − *u*(*t*) is defined as synchronization error. For the OPCL synchronization to be achieved, the error must tend to zero and it can be verified by Taylor’s series [23]. If the real parts of the eigenvalues from *H* are negative, the synchronization will be successful. This is a necessary condition but not enough since there may be an *H* with eigenvalues equal to zero and the synchronization can occur.

Again, lets us consider Sprott’s case L. The master system is proposed being similar to the original system. The open loop part in the slave system is null (*D*_1_(*u*(*t*)) = 0). For the closed part, the master system partial derivative is given in (24), and *H* is proposed in (25), where *P* is a constant value and depending on how many values are proposed, it will be the complexity to obtain the closed loop part. The eigenvalues of *H* determine that P must be negative. For example: if *P* = −3, the eigenvalues shown in (26) have real part negative and thereby the condition described above is accomplished. Therefore, the closed loop contribution is given in (27). Finally, with the open-closed loop contribution, the chaotic slave oscillator for Sprott’s case L is given in (28).

$$\begin{array}{c} \frac{\delta }{\delta t}F\left(u\left(t\right)\right)=(\begin{array}{ccc} 0 & 1 & 3.9\\ 1.8x_{m1} & -1 & 0\\ -1 & 0 & 0 \end{array}) \end{array}$$ (24)

$$\begin{array}{c} H=(\begin{array}{ccc} 0 & 1 & 3.9\\ P & -1 & 0\\ -1 & 0 & 0 \end{array}) \end{array}$$ (25)

$$\begin{array}{c} \begin{array}{cc} & \lambda_{1}=-0.5858\\ & \lambda_{2}=-0.207-j2.57\\ & \lambda_{3}=-0.207+j2.57 \end{array} \end{array}$$ (26)

$$\begin{array}{c} \begin{array}{cc} & D_{2}=((\begin{array}{ccc} 0 & 1 & 3.9\\ P & -1 & 0\\ -1 & 0 & 0 \end{array})-(\begin{array}{ccc} 0 & 1 & 3.9\\ 1.8x_{m1} & -1 & 0\\ -1 & 0 & 0 \end{array}))\left(v_{t}-u_{t}\right)\\ & \;=(\begin{array}{c} 0\\ \left(P-1.8\right)\left(x_{s1}-x_{m1}\right)\\ 0 \end{array}) \end{array} \end{array}$$ (27)

$$\begin{array}{c} \frac{d}{dt}v\left(t\right)=\{\begin{array}{c} x_{s2}+x_{s3}\\ 0.9x_{s1}^{2}-x_{s2}+\left(P-1.8\right)\left(x_{s1}-x_{m1}\right)\\ 1-x_{s1} \end{array};P<0 \end{array}$$ (28)

**Fig 7:**
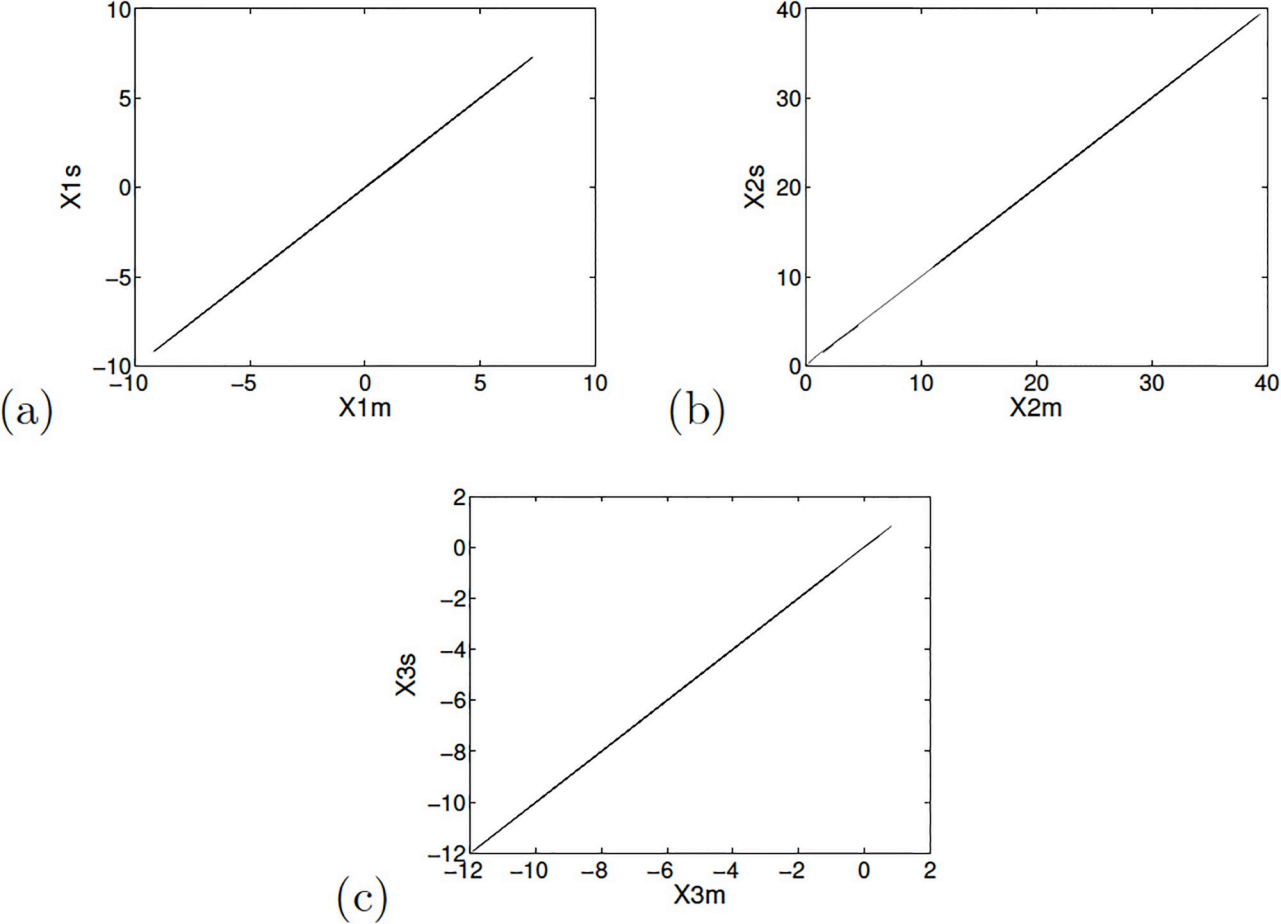
Phase diagrams for the master and slave state variables: (a) *x*_1_, (b) *x*_2_ and (c) *x*_3_ for Sprott’s case L applying OPCL technique with *P* = −3 in (25).

**Fig 8:**
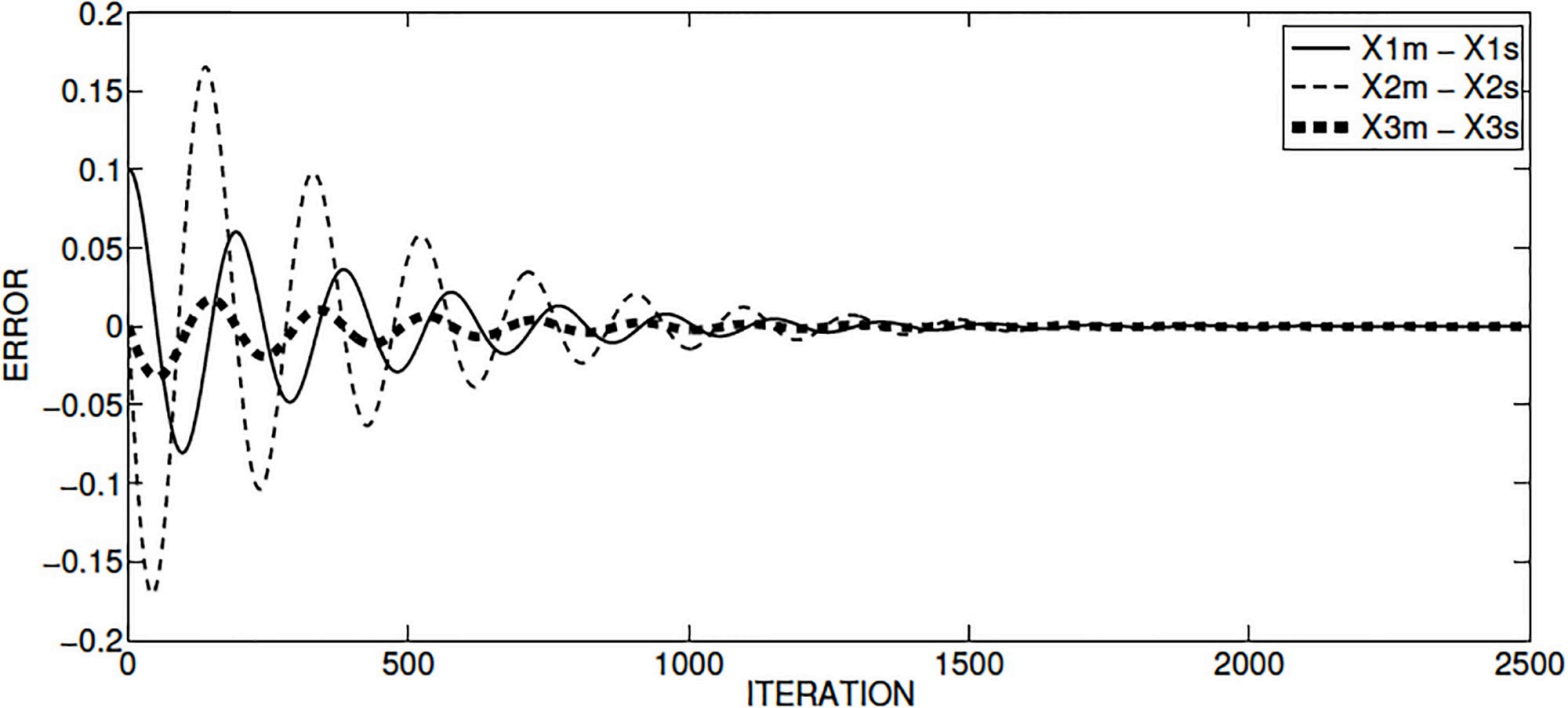
Synchronization error of Sprott’s case L, for the master and slave state variables applying OPCL technique.

The synchronization between the master and slave chaotic oscillators is shown in Fig 7. The synchronization errors are shown in Fig 8, where it is observed that the minimum error occurs around iteration 2000.

## 4 FPGA-based implementation of a chaotic secure communication system

We selected the six chaotic oscillators providing high positive Lyapunov exponent values, they are: the chaotic oscillator based on SNLF series, and Sprott’s cases G and L from Table 4, and the chaotic oscillator based on Negative Slopes, and Sprott’s cases B and S from Table 5. The electronic implementations of these chaotic oscillators were performed herein by exploiting the advantages of the field-programmable gate arrays (FPGAs) for fast prototyping [32]. As already detailed in [33], the mathematical equations modeling a chaotic oscillator can be described through the hardware description language (HDL), which in this work we use the tool Active-HDL. In this manner, the chaotic oscillators are implemented with adders, subtractors and multipliers, as detailed in [32]. In addition, a PWL function having the forms shown in Fig 1 can be implemented with comparators. The HDL code can be generated according to [33], where it is highlighted that the size of the digital blocks require the number of bits being used, and in this case we use fixed-point notation with format 7.21.

The FPGA-based implementations were performed using Cyclone IV GX EP4CGX150DF31C7 from ALTERA. For example: The descriptions of the digital blocks for the chaotic oscillator based on SNLF series and Sprott’s cases G and L, were performed in two ways called: Type A and type B. All the blocks of Type A include a clock-pin CLK, so that all of them are sequential. The descriptions classified as Type B do not include CLK, so that they are combinational. Table 6 lists the FPGA resources of the chaotic oscillators with the highest positive Lyapunov exponent. Three one-step methods were applied for each chaotic oscillator, and the FPGA implementations are of type A and B. We also list the maximum frequency response of the blocks that is provided by the FPGA synthesizer. The maximum frequency is multiplied by the number of clock cycles that are required to process the data from the input to the output, so that the processing speed or latency is listed in the last column in nanoseconds (ns).

**Table 6:** FPGA resources of the chaotic oscillator based on SNLF series, and Sprott’s cases G and L by applying three numerical methods and using Cyclone IV GX EP4CGX150DF31C7.

*Oscillator*	*Numerical Method*	*Block Type*	*Logic Elements*	*Registers*	*Maximun Frequency (MHz)*	*Cycles*	*Iteration Latency (ns)*
***SNLF Series***	Forward-Euler	A	911	592	116.04	9	77.58
		B	811	118	33.48	2	60
	Trapezoidal	A	4539	906	66.67	12	179
		B	4507	118	18.00	2	111
	4th-order Runge-Kutta	A	4745	1106	67.53	17	251
		B	4686	118	14.00	2	143
***Sprott’s Case G***	Forward-Euler	A	649	411	113.8	8	70
		B	551	104	39.2	2	51
	Trapezoidal	A	1314	603	111.6	12	108
		B	1203	104	23.1	2	87
	4th-order Runge-Kutta	A	2024	1191	106.7	16	150
		B	1918	104	19.8	2	101
***Sprott’s Case L***	Forward-Euler	A	661	417	116.3	8	69
		B	557	104	37.4	2	53
	Trapezoidal	A	1386	618	109.8	12	109
		B	1256	104	30.9	2	65
	4th-order Runge-Kutta	A	2113	1219	115.42	17	147
		B	2008	104	19.1	2	105

**Fig 9:**
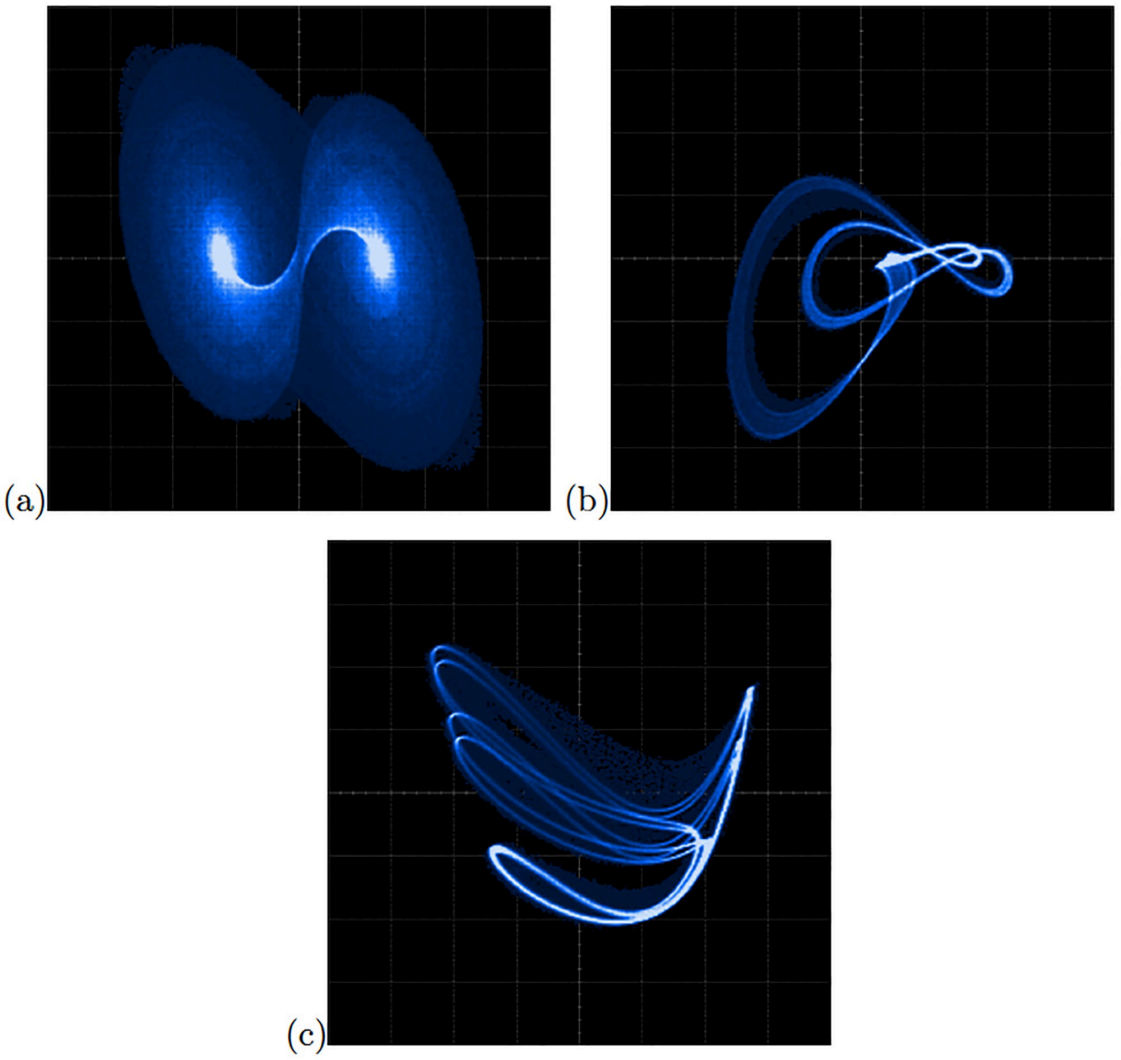
Experimental phase-space portraits *x*_1_ − *x*_2_ of the chaotic oscillators: (a) Based on SNLF series with axes *X* = 2*V*/*div* and *Y* = 1*V*/*div*, (b) Sprott’s case G with axes *X* = 1*V*/*div* and *Y* = 1*V*/*div*, and (c) Sprott’s case L with axes *X* = 1*V*/*div* and *Y* = 1*V*/*div*.

As one sees, the sequential or type A blocks require more resources than the type B. In the same manner, as the 4th-order Runge-Kutta numerical method requires more resources than Forward Euler and the Trapezoidal methods, those implementations have slower time response, as shown in the last column in Table 6. Thus, the maximum processing speed or latency is accomplished by using descriptions of type B (combinational blocks). Fig 9(a) shows the experimental observation of a 2-scroll attractor plotting the state variables *x*_1_-*x*_2_ for the oscillator based on SNLF series. Fig 9(b) shows the phase portraits of Sprott’s collection case G, and Fig 9(c) shows the attractor of Sprott’s case L.

**Fig 10:**
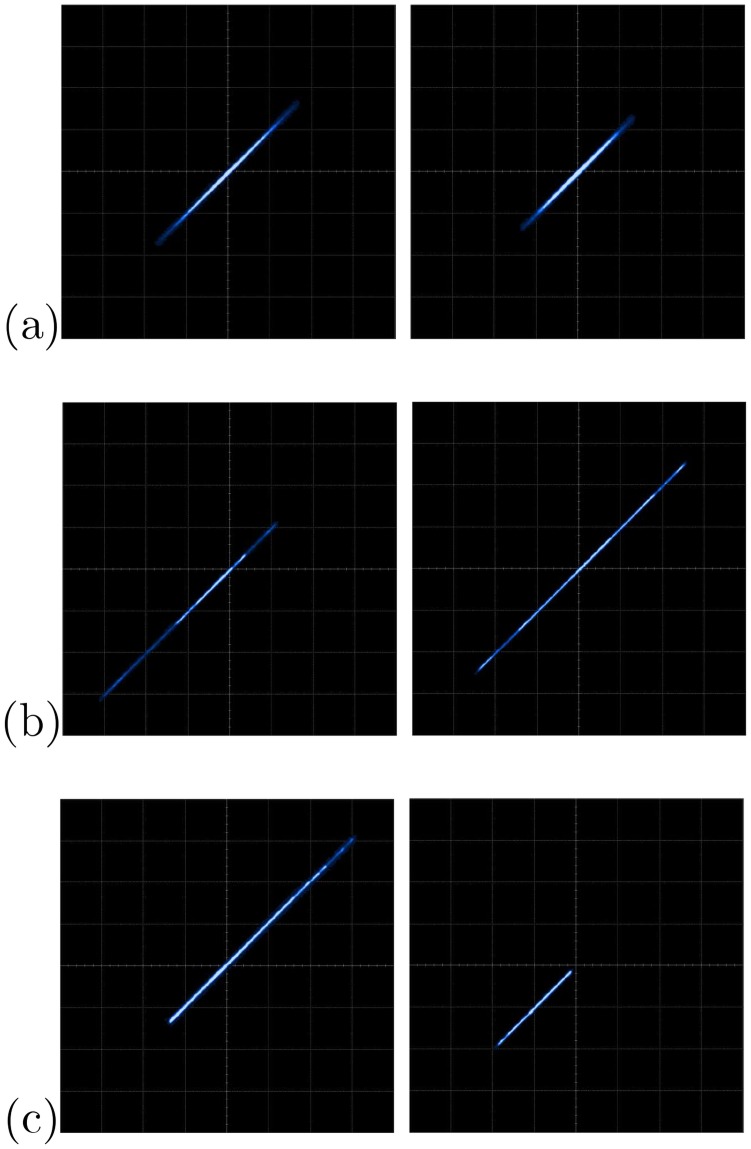
Experimental synchronization of *x*_2_ (left column) and *x*_3_ (right column) phase diagrams applying Pecora-Carroll synchronization technique to the chaotic oscillator: (a) based on SNLF series, (b) Sprott’s case G, and (c) Sprott’s case L. In all cases with axes channels: *X* = 1*V*/*div* and *Y* = 1*V*/*div*.

**Fig 11:**
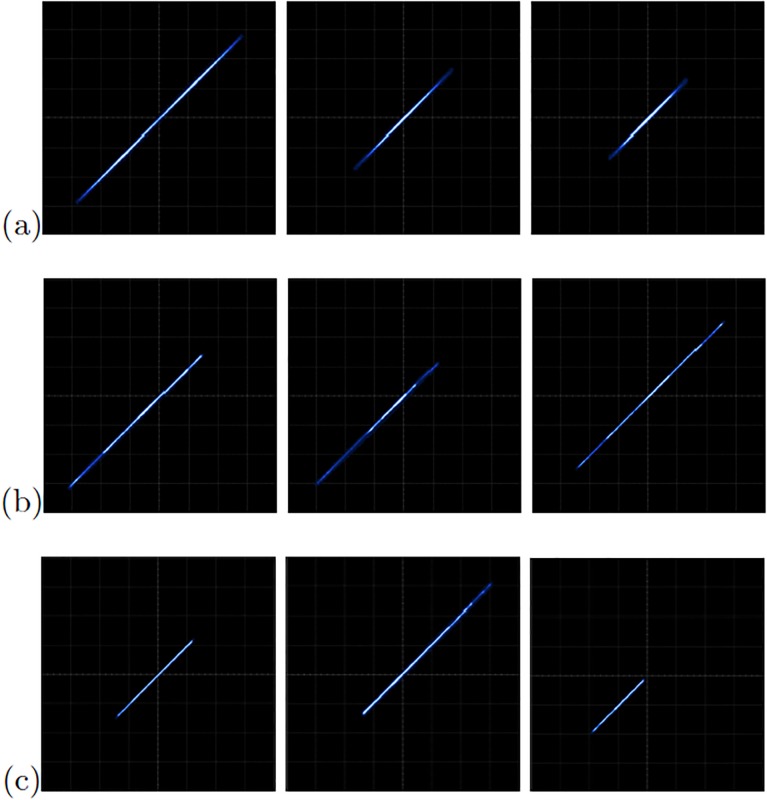
Experimental synchronization of *x*_1_ (left column), *x*_2_ (center column), and *x*_3_ (right column) phase diagrams applying Hamiltonian forms to the chaotic oscillator: (a) based on SNLF series, (b) Sprott’s case G, and (c) Sprott’s case L. In all cases with axes channels: *X* = 1*V*/*div* and *Y* = 1*V*/*div*.

**Fig 12:**
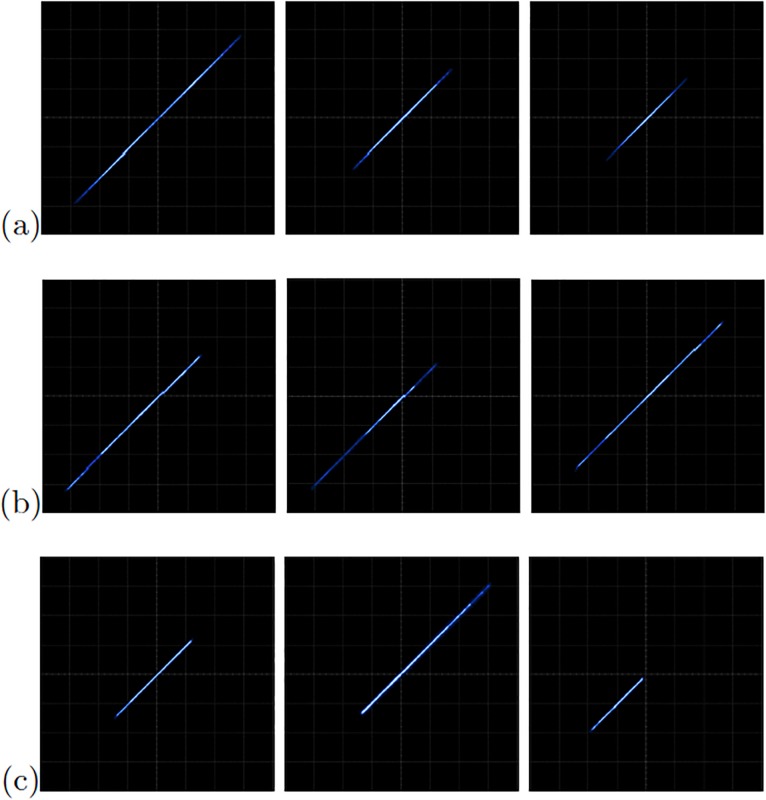
Experimental synchronization of *x*_1_ (left column), *x*_2_ (center column), and *x*_3_ (right column) phase diagrams applying OPCL to the chaotic oscillator: (a) based on SNLF series, (b) Sprott’s case G, and (c) Sprott’s case L. In all cases with axes channels: *X* = 1*V*/*div* and *Y* = 1*V*/*div*.

The FPGA-based implementations of the chaotic oscillator based on Negative Slopes, and Sprott’s cases B and S, where performed in the same way. Further, the six chaotic oscillators were synchronized in a master-slave topology applying the three synchronization techniques. Lets us consider first the synchronization of the experimental chaotic attractors from Fig 9. Applying the Pecora-Carroll synchronization technique, the implementation accomplish the diagram shown in Fig 2, where *x*_1_ is the driver. The experimental results were observed in an oscilloscope and are shown in Fig 10. The experimental results of the synchronization applying Hamiltonian forms and OPCL techniques are shown in Figs 11 and 12, respectively.

Table 7 lists the FPGA resources of these chaotic oscillators using Cyclone IV GX EP4CGX150DF31C7. It can be observed that the FPGA-based implementation of the Pecora-Carroll synchronization technique requires less logical elements and registers than the other synchronization techniques, but it uses twice the number of clock cycles per iteration. On the other hand, the synchronization techniques with Hamiltonian forms and OPCL show similar resources characteristics. Besides, Hamiltonian forms technique is slightly faster than OPCL.

**Table 7:** Resources for the three synchronization techniques applied to the chaotic oscillator based on SNLF series, and Sprott’s cases G and L, using the FPGA Cyclone IV GX EP4CGX150DF31C7.

*Synchronization Technique*	*Chaotic Oscillator*	*Logic Elements*	*Registers*	*Maximun Frequency (Mhz)*	*Cycles*	*Iteration Latency (ns)*
***Pecora-Carroll***	SNLF series	7391	1936	103.4	40	387
	Sprott’s case G	3131	1763	108.31	36	332
	Sprott’s case L	3350	1919	115.83	40	345
***Hamiltonian Forms***	SNLF series	9781	2628	68.17	17	249
	Sprott’s case G	4348	2678	107.87	17	158
	Sprott’s case L	4458	2732	113.06	20	177
***OPCL***	SNLF series	9894	2688	66.26	18	272
	Sprott’s case G	4430	2680	108.52	18	166
	Sprott’s case L	4682	2620	72.35	21	290

**Fig 13:**
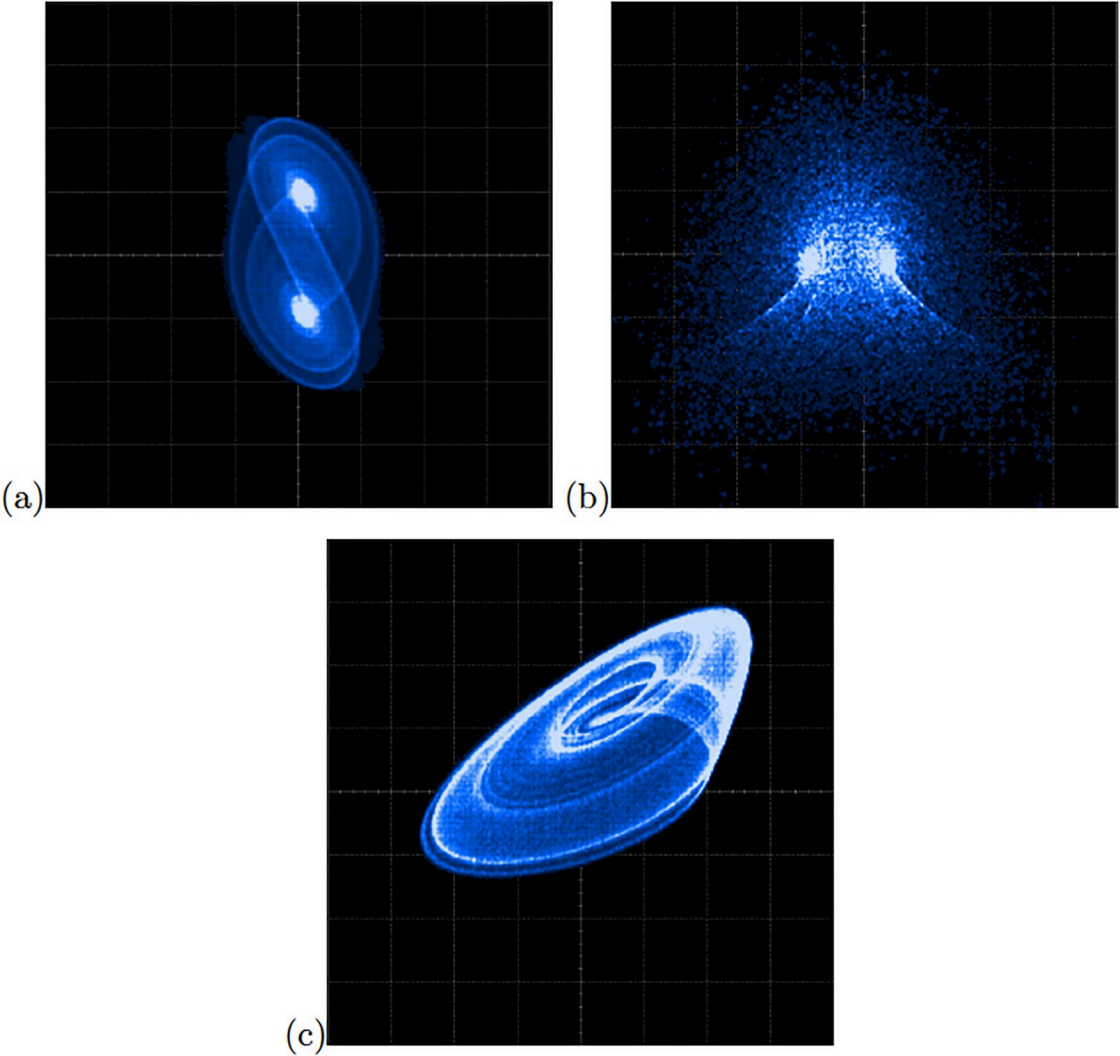
Experimental phase-space portraits *x*_1_ − *x*_2_ of the chaotic oscillators: (a) Based on Negative Slopes, (b) Sprott’s case B, and (c) Sprott’s case S. In all cases with axes X = 1V = div and Y = 1V = div.

**Fig 14:**
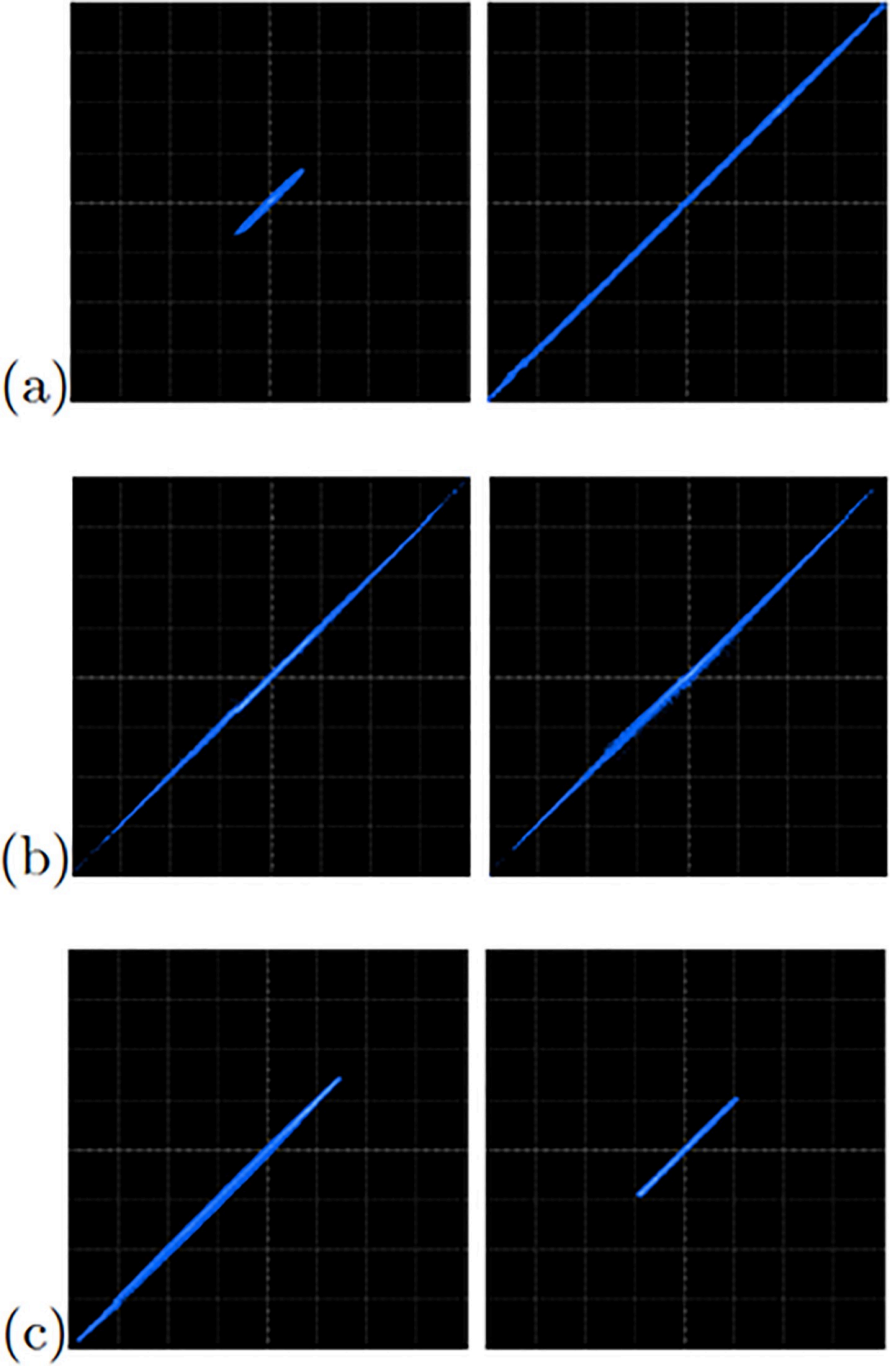
Experimental phase diagrams applying Pecora-Carroll synchronization technique to the chaotic oscillator: (a) based on Negative Slopes (*x*_2_ = left column and *x*_3_ = right column), (b) Sprott’s case B (*x*_1_ = left column and *x*_3_ = right column), and (c) Sprott’s case S (*x*_1_ = left column and *x*_2_ = right column). In all cases with axes channels: X = 2V = div and Y = 2V = div.

**Fig 15:**
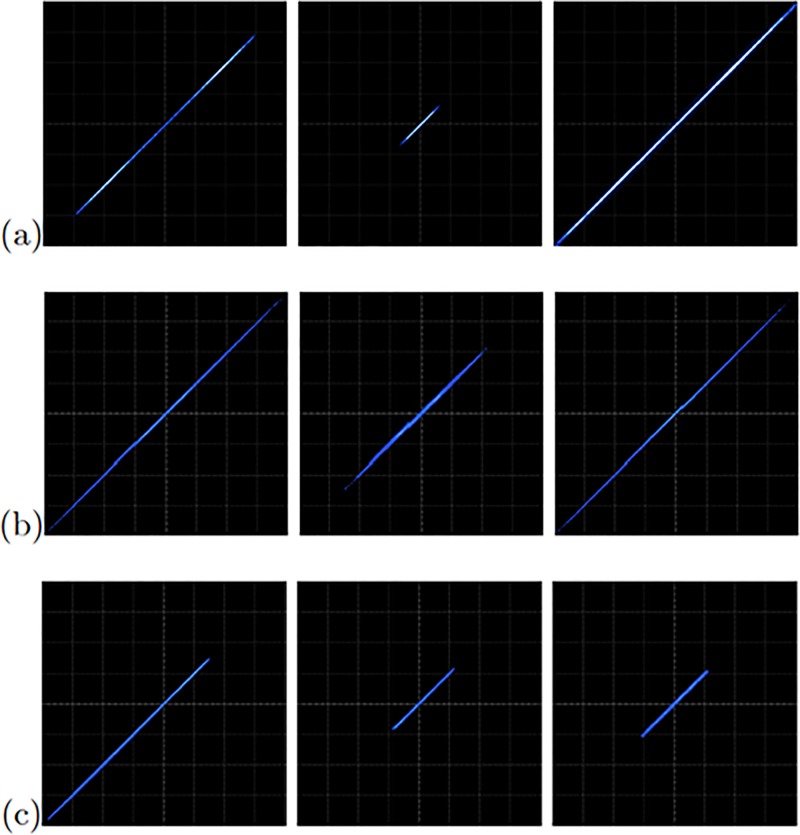
Experimental phase diagrams of the master-slave state variables *x*_1_ (left column), *x*_2_ (center column), and *x*_3_ (right column), applying Hamiltonian forms to the chaotic oscillator: (a) based on Negative Slopes, (b) Sprott’s case B, and (c) Sprott’s case S. In all cases with axes channels: X = 2V = div and Y = 2V = div.

**Fig 16:**
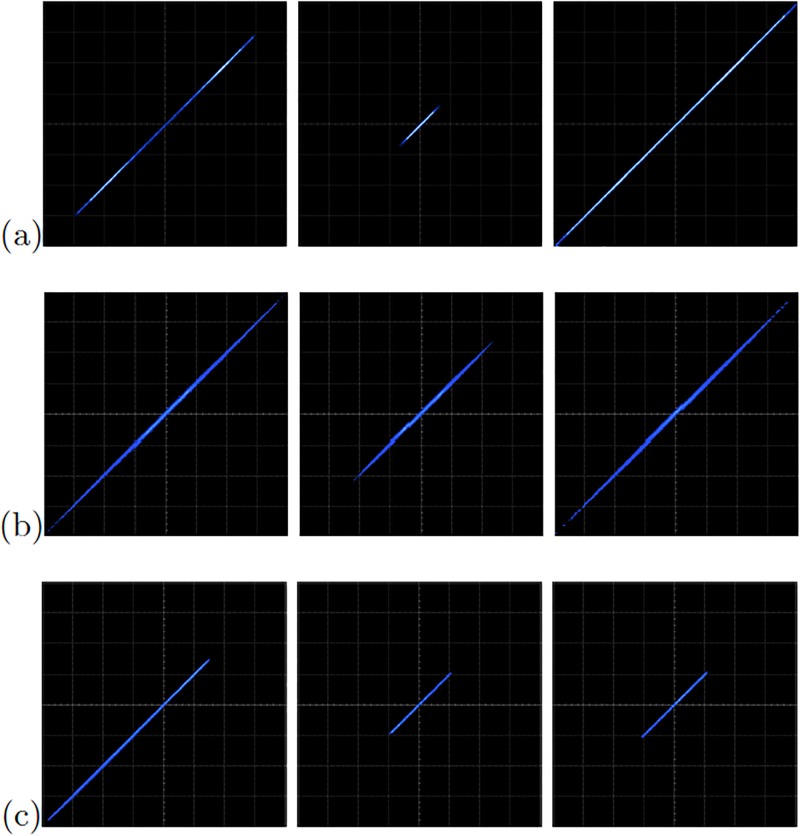
Experimental phase diagrams of the master-slave state variables *x*_1_ (left column), *x*_2_ (center column), and *x*_3_ (right column), applying OPCL to the chaotic oscillator: (a) based on Negative Slopes, (b) Sprott’s case B, and (c) Sprott’s case S. In all cases with axes channels: X = 2V = div and Y = 2V = div.

Fig 13(a) shows the experimental observation of a 2-scroll attractor plotting the state variables *x*_1_-*x*_2_ for the oscillator based on Negative Slopes. Fig 13(b) shows the phase portraits of Sprott’s case B, and Fig 13(c) shows the attractor of Sprott’s case L. Using these chaotic oscillators, we performed their master-slave synchronization. In this manner, the synchronization applying Pecora-Carroll technique is shown in Fig 14, for the chaotic oscillator based on Negative Slopes having *x*_1_ as driver. The Sprott’s cases B and S have *x*_2_ and *x*_3_ as driver, respectively. Hamiltonian forms and OPCL synchronization techniques are shown in Figs 15 and 16, respectively.

**Fig 17:**
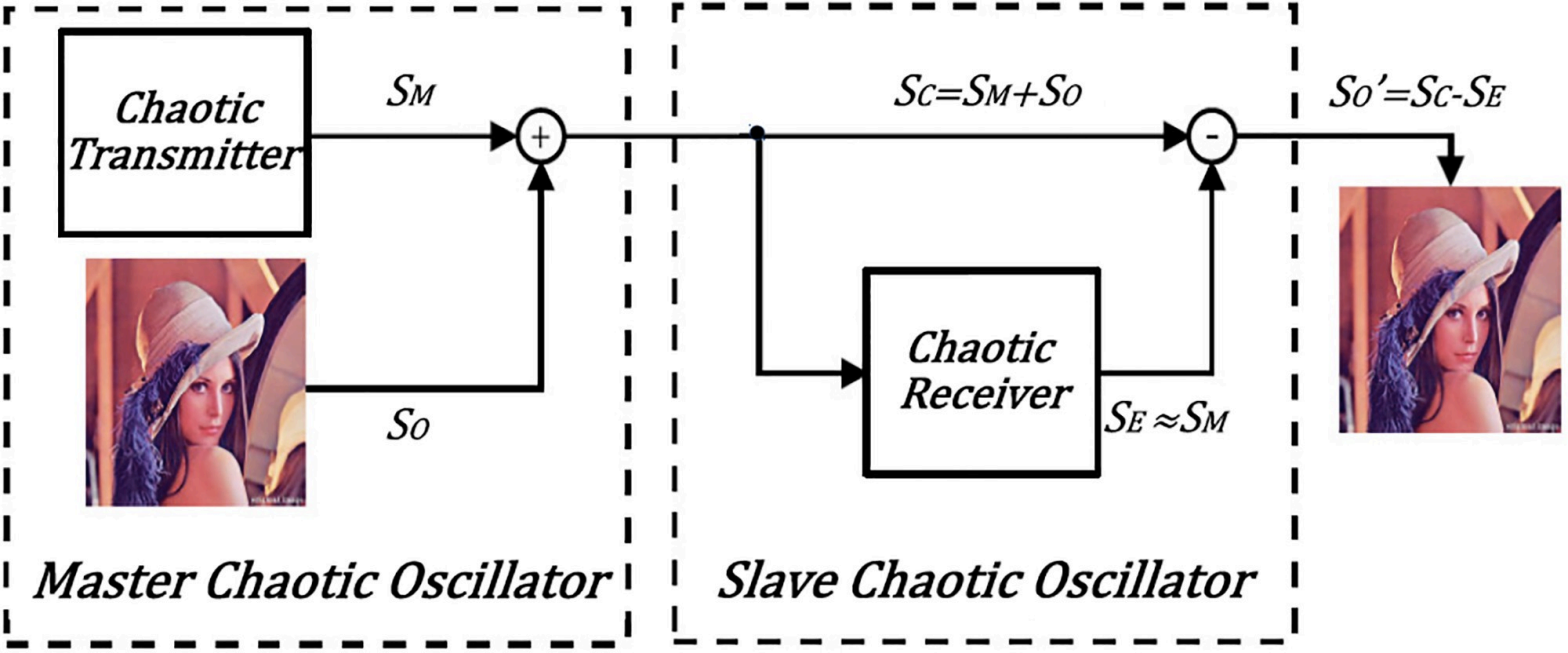
Chaotic secure communication system based on the master-slave topology.

The FPGA-based implementation of the three synchronization techniques using the six chaotic oscillators with the high positive Lyapunov exponent values are used herein to implement a chaotic secure communication system to transmit an image. The communication system requires modulation and demodulation blocks that can be implemented as addition and subtraction operations for chaotic masking, as shown in Fig 17. The master oscillator is located in the transmitter block to mix the original image (*S*_*O*_) with chaos (*S*_*M*_). Later, the encrypted image (*S*_*C*_) is send through a chaotic channel, and then at the receiver block the slave oscillator (*S*_*E*_), which behaves as the master, subtract chaos (*S*_*M*_) to recover the original data ($S_{O}^{\prime }$). If exact synchronization is accomplished, then the error between the original and the recovered image will be zero, otherwise the communication system presents loss of information equal to *S*_*C*_ − *S*_*E*_.

**Fig 18:**
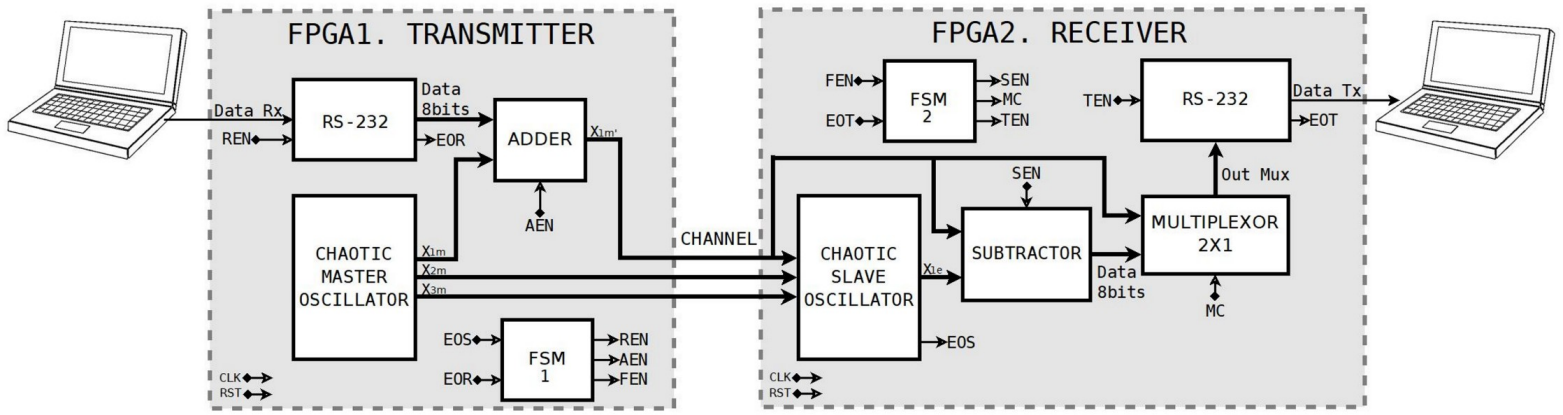
Block diagram of the implementation of a chaotic secure communication system based on a master-slave topology.

The image being transmitted is of RGB type and has a size of 256×256 pixels. It is send to the FPGA-based chaotic secure communication system from a personal computer (PC) through the RS-232 protocol. Fig 18 shows the block diagram of the interconnections among the PC and two FPGAs during the image transmission process. The FPGA1 contains the transmission part and the finite state machine (FSM1 block) controls it, EOS activates the system when the master-slave synchronization (chaotic oscillator’s blocks) is successful. Then REN enables the RS-232 receiver block, any data can be received serially and when the reception finishes, EOR is activated. Then, FSM enables the adder block with the AEN signal and sends the contaminated data (original image + chaos) to the FPGA2, which contains the receiver (slave chaotic oscillator) block that is controlled by the finite state machine (FSM2 block) enabled with the FEN signal. When the contaminated data is in the PFGA2, the subtractor block is activated with SEN signal, which is responsible for recovering the original data. Finally, the multiplexer block is enabled with MC to transmit the contaminated and recovered data through the RS-Transmitter block controlled by the TEN signal to the computer. When the transmission finishes, EOT is activated and the system is ready to receive new data.

**Fig 19:**
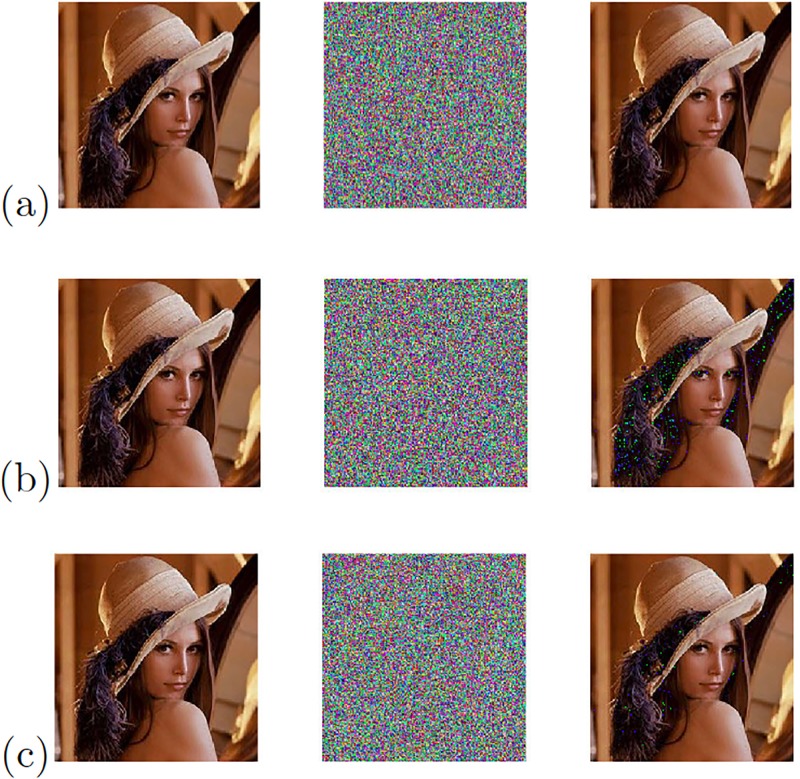
Original (left column), encrypted (center column), and recovered (right column) images applying Pecora-Carroll synchronization technique to the chaotic oscillator: (a) Based on SNLF series, (b) Sprott’s case G, and (c) Sprott’s case L.

Fig 19 shows the experimental results of the image transmission, when applying the Pecora-Carroll synchronization technique to the chaotic oscillator Based on SNLF series, and Sprott’s cases G and L, for which their positive Lyapunov exponent were evaluated using “TISEAN 3.0.1”. The errors that are appreciated in the recovered image are due to the lack of accuracy of the Pecora-Carroll synchronization technique.

**Fig 20:**
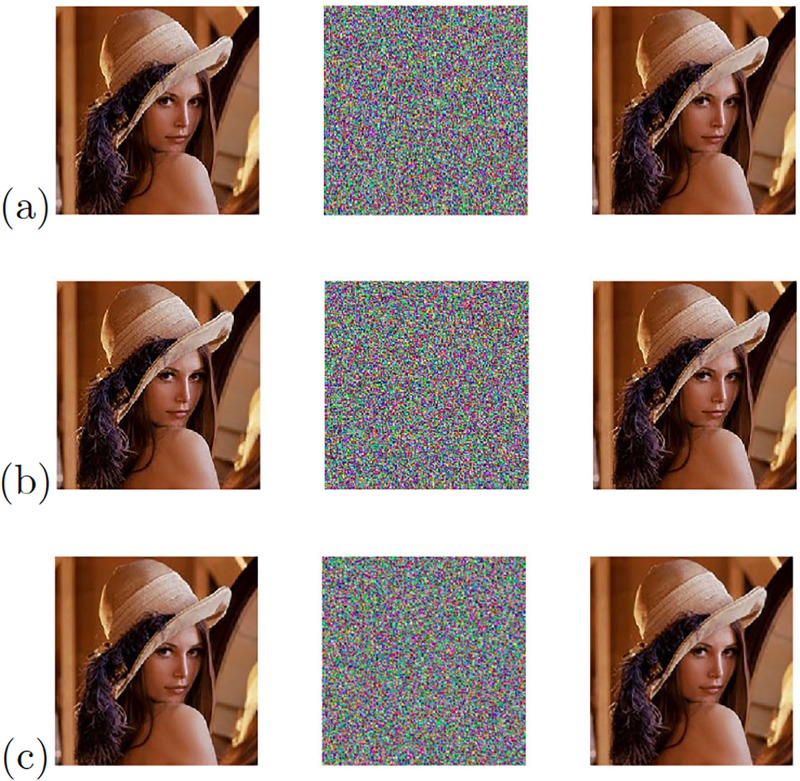
Original (left column), encrypted (center column), and recovered (right column) images applying Hamiltonian forms to the chaotic oscillator: (a) Based on SNLF series, (b) Sprott’s case G, and (c) Sprott’s case L.

**Fig 21:**
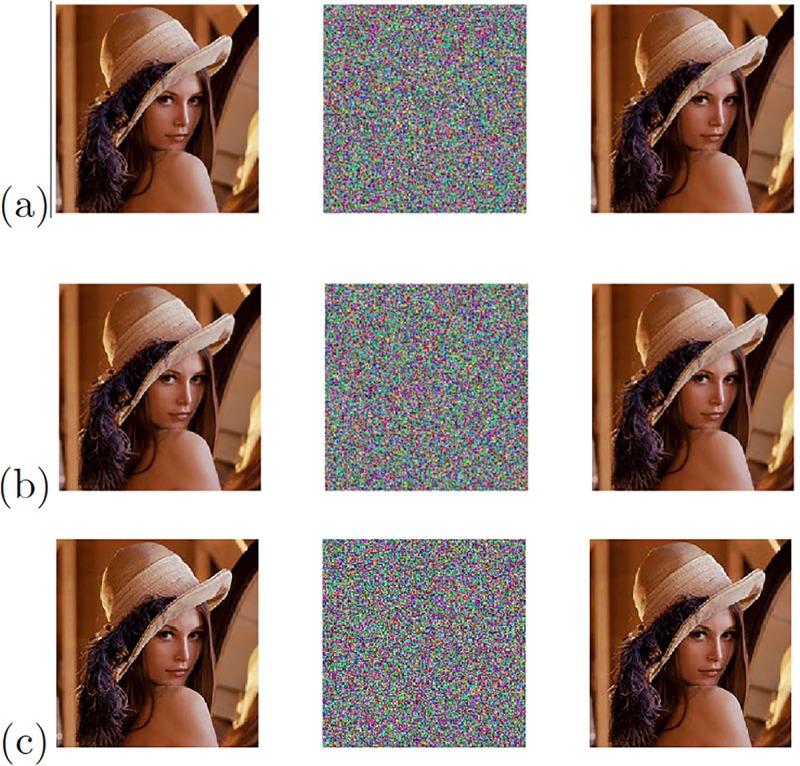
Original (left column), encrypted (center column), and recovered (right column) images applying OPCL technique to the chaotic oscillator: (a) Based on SNLF series, (b) Sprott’s case G, and (c) Sprott’s case L.

The chaotic secure communication system is also implemented using the synchronization technique based on Hamiltonian forms and the blocks shown in Fig 17. Fig 20 shows the experimental results of the image transmission. In the same manner, Fig 21 shows the experimental results of the image transmission using the OPCL synchronization technique.

The experimental results provided above are summarized in Table 8, showing the correlations between the original image and the chaotic channel, and between the original and the recovered images. The three chaotic oscillators with the high positive Lyapunov exponent values from Table 4 were used in the three synchronization techniques, and under the same conditions. Table 8 shows that the Pecora-Carroll synchronization technique cannot recover the original image as it is done by applying the other synchronization techniques based on Hamiltonian forms and OPCL. According to the correlation between the original image and the chaotic channel, the best synchronization technique is the one based on OPCL with Sprott’s case L. It can also be observed that the chaotic oscillators based on SNLF series and Sprott’s case L use the state variable *x*_1_, and Sprott’s case G uses *x*_3_ because it provided the highest positive Lyapunov exponent value.

**Table 8:** Correlations among the original, encrypted and recovered data using the three synchronization techniques and the oscillators with the highest positive Lyapunov exponent obtained with “TISEAN 3.0.1”.

*Synchronization Technique*	*Chaotic Oscillator*	*Transmission Variable*	*Transmission Correlation* $\frac{Original\;Img.+Chaotic\;Signal}{Original\;Img.}$	*Recovery Correlation* $\frac{Original\;Img.}{Recovery\;Img.}$
***Pecora & Carroll***	SNLF series	*x*_1_	-0.00083	0.9999
	Sprott’s Case G	*x*_3_	0.0075	0.9808
	Sprott’s Case L	*x*_1_	-0.0165	0.9934
***Hamiltonian Forms***	SNLF series	*x*_1_	0.00002	1
	Sprott’s Case G	*x*_3_	0.0045	1
	Sprott’s Case L	*x*_1_	0.0005	1
***OPCL***	SNLF series	*x*_1_	0.0015	1
	Sprott’s Case G	*x*_3_	0.0031	1
	Sprott’s Case L	*x*_1_	-0.00059	1

**Fig 22:**
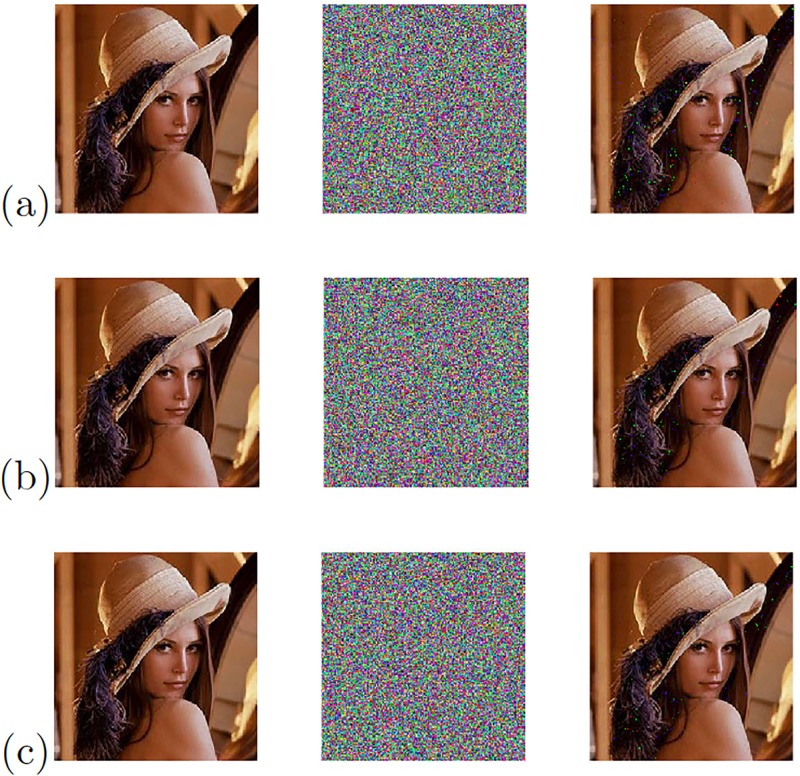
Original (left column), encrypted (center column), and recovered (right column) images applying Pecora-Carroll synchronization technique to the chaotic oscillator: (a) Based on Negative Slopes (Variable *x*_2_), (b) Sprott’s case B (Variable *x*_1_), and (c) Sprott’s case S (Variable *x*_1_).

**Fig 23:**
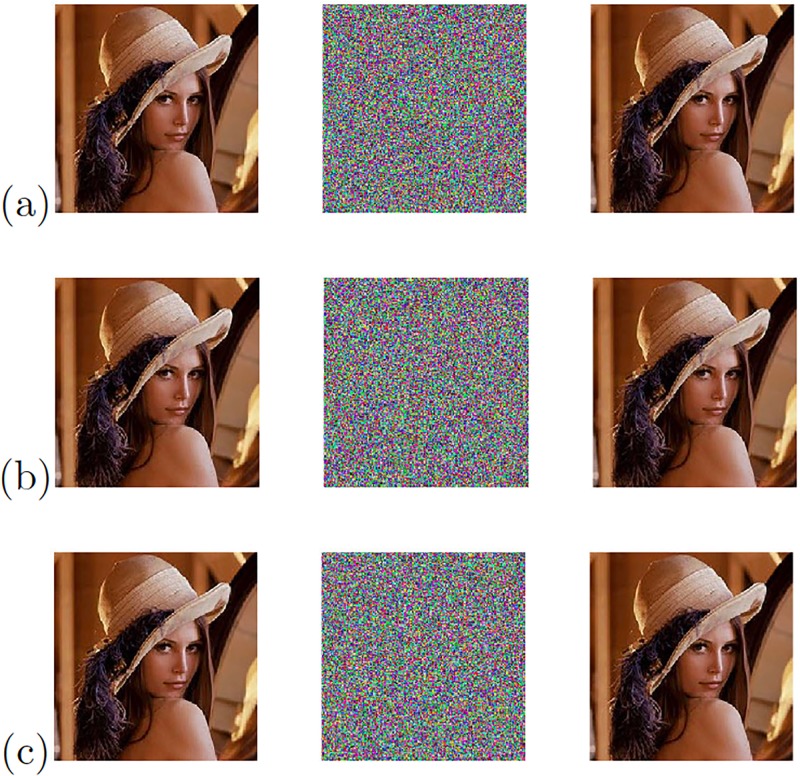
Original (left column), encrypted (center column), and recovered (right column) images applying Hamiltonian forms to the chaotic oscillator: (a) Based on Negative Slopes (Variable *x*_1_), (b) Sprott’s case B (Variable *x*_1_), and (c) Sprott’s case S (Variable *x*_2_).

**Fig 24:**
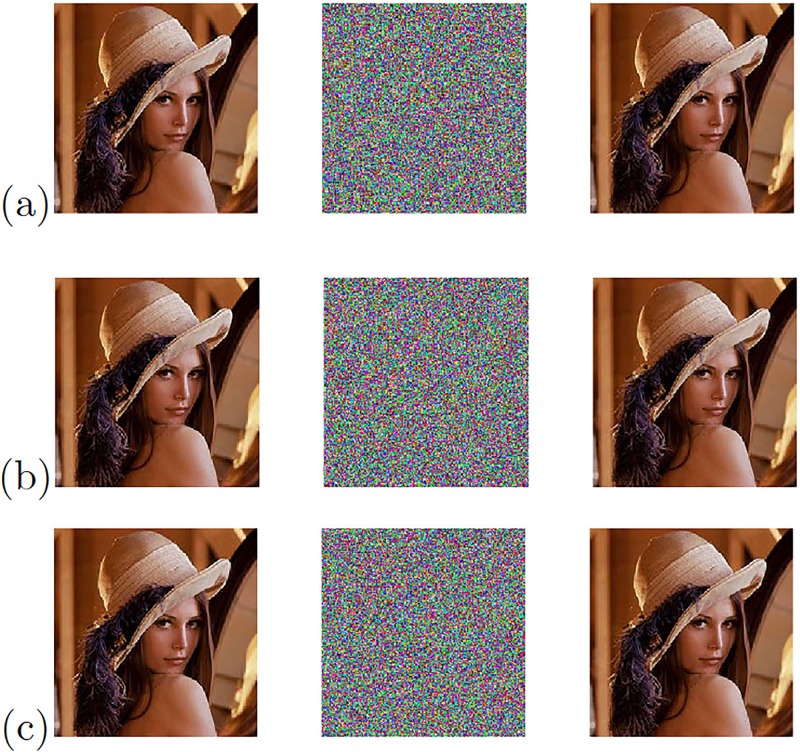
Original (left column), encrypted (center column), and recovered (right column) images applying OPCL to the chaotic oscillator: (a) Based on Negative Slopes (Variable *x*_3_), (b) Sprott’s case B (Variable *x*_1_), and (c) Sprott’s case S (Variable *x*_3_).

The remaining three chaotic oscillators with the high positive Lyapunov exponent values from Table 5 provided the experimental results shown in Fig 22 when applying the Pecora-Carroll synchronization technique. Fig 23 shows the chaotic secure communication system implemented using the synchronization technique based on Hamiltonian forms and Fig 24 shows the experimental results of the image transmission using the OPCL synchronization technique.

In theses cases, the experimental results are summarized in Table 9, showing the correlations between the original image and the chaotic channel, and between the original and the recovered image. In these cases, the transmission was implemented using the three state variables of the chaotic oscillators, and as shown above from Table 5, applying “Wolf’s algorithm” leads us to obtain the highest positive Lyapunov exponent value. In the same manner, Table 9 shows that the Pecora-Carroll synchronization technique cannot recover the original image, and the transmission when using the chaotic oscillators for Sprott’s cases B (Variable *x*_3_) and S (variable *x*_2_) is unsuccessful because the synchronization error is very high. On the other hand, when applying the synchronization techniques based on Hamiltonian forms and OPCL, the recovery of the original image is quite successful even when using all the state variables. From Table 9, and according to the correlation between the original image and the chaotic channel, the best synchronization technique is the one based on OPCL with Negative Slopes and Sprott’s case B using the state variable *x*_1_.

**Table 9:** Correlations among the original, encrypted and recovered data using the three synchronization techniques and the oscillators with the high positive Lyapunov exponent values obtained with “Wolf’s algorithm”.

*Synchronization Technique*	*Chaotic Oscillator*	*Transmission Variable*	*Transmission Correlation* $\frac{Original\;Img.+Chaotic\;Signal}{Original\;Img.}$	*Recovery Correlation* $\frac{Original\;Img.}{Recovery\;Img.}$
***Pecora & Carroll***	Neg. Slopes PWL	*x*_1_	Driver	Driver
		*x*_2_	-0.0045	0.9815
		*x*_3_	-0.0068	0.9841
	Sprott’s Case B	*x*_1_	0.0068	0.9840
		*x*_2_	Driver	Driver
		*x*_3_	High Error	High Error
	Sprott’s Case S	*x*_1_	0.0023	0.9913
		*x*_2_	High Error	High Error
		*x*_3_	Driver	Driver
***Hamiltonian Forms***	Neg. Slopes PWL	*x*_1_	-0.0007	1
		*x*_2_	0.0022	1
		*x*_3_	-0.0011	1
	Sprott’s Case B	*x*_1_	0.0007	1
		*x*_2_	-0.0015	1
		*x*_3_	0.0009	1
	Sprott’s Case S	*x*_1_	-0.0012	1
		*x*_2_	-0.0011	1
		*x*_3_	0.0015	1
***OPCL***	Neg. Slopes PWL	*x*_1_	-0.0035	1
		*x*_2_	-0.0050	1
		*x*_3_	-0.0013	1
	Sprott’s Case B	*x*_1_	0.0001	1
		*x*_2_	0.0023	1
		*x*_3_	-0.0027	1
	Sprott’s Case S	*x*_1_	0.0014	1
		*x*_2_	-0.0023	1
		*x*_3_	-0.0012	1

## 5 Conclusion

This article showed that the value of the positive Lyapunov exponent matters when looking for the implementation of a chaotic secure communication system for image transmission. That way, we analyzed 22 chaotic oscillators, three based on PWL functions and nineteen from Sprott’s collection. Each chaotic oscillator was characterized evaluating its positive Lyapunov exponent and Kaplan-York dimension using the free-available software TISEAN and applying “Wolf’s algorithm”. Since TISEAN requires time series data, we selected the three chaotic oscillators providing the high positive Lyapunov exponent values, and also we selected the three chaotic oscillators that when applying “Wolf’s algorithm”, they provided the high positive Lyapunov exponent values. The six chaotic oscillators were different and they were synchronized applying the seminal work of Pecora-Carroll, Hamiltonian forms, and OPCL techniques. The synchronization errors and the latency of each case were measured and experimental results were listed by using FPGAs to implement chaotic secure communication systems using the six chaotic oscillators. A personal computer was used to pass an image to the chaotic transmitter block and to recover the image at the recover block. The correlations among the original, the contaminated and the recovered images were evaluated observing that the image transmission is very successful and faster when the error and the correlation between the image and the chaotic channel are minimum. Hamiltonian forms provided the lowest latency in all cases and lowest correlation between the image and chaotic channel. The final conclusion is that using chaotic oscillators with high positive Lyapunov exponent value and synchronization techniques providing low error guarantee high security in a communication system, and this system is suitable for FPGA implementation.
